# Molecular Dynamics Simulation of the Aggregation Behavior of Typical Aromatic Pollutants and Its Influence on the *n*-Octanol–Air Partition Coefficient

**DOI:** 10.3390/toxics13090721

**Published:** 2025-08-28

**Authors:** Wanran Li, Wencong Fan, Jing Zhang, Shuhua Chen, Yawei Shi, Guanghui Ding

**Affiliations:** 1 College of Intelligence and Electronic Engineering, Dalian Neusoft University of Information, Dalian 116023, China; liwanran@neusoft.edu.cn; 2 College of Environmental Science and Engineering, Dalian Maritime University, Linghai Road 1, Dalian 116026, China; fanwencong@dlmu.edu.cn (W.F.); ywshi@dlmu.edu.cn (Y.S.); 3 College of Environment and Chemical Technology, Dalian University, Dalian 116622, China; zhangjing@dlu.edu.cn (J.Z.); chenshuhua@dlu.edu.cn (S.C.)

**Keywords:** aromatic pollutants, aggregation behavior, *n*-octanol–air partition coefficient, π–π interactions, molecular dynamics simulation

## Abstract

The aggregation behavior of typical aromatic pollutants in the *n*-octanol phase and its influence on the *n*-octanol–air partition coefficient (*K*_OA_) were investigated using molecular dynamics simulation. The aggregate proportion of selected aromatic pollutants gradually increased with increasing simulation time and then reached a dynamic equilibrium state. It is interesting to find that the higher the concentration of aromatic pollutants, the more aggregates formed in the *n*-octanol phase. Log *K*_OA_ values of these aromatic pollutants were subsequently estimated based on the percentages of aggregates and the solvation free energy from the gas phase to the *n*-octanol phase. The log *K*_OA_ values were also found to gradually increase with increasing concentration. Therefore, the effect of concentration on *K*_OA_ should be taken into consideration during the analysis of the environmental behavior and transport of these aromatic pollutants. In addition, it was found that π–π interactions drive the formation of different numbers of aggregates for different aromatic pollutants, a phenomenon that affects the *K*_OA_ values of aromatic pollutants. The above results shed some light on the effects of aggregates and concentration on the partition behavior of aromatic pollutants and provide a theoretical basis for the correction of *K*_OA_ of aromatic pollutants in the environment.

## 1. Introduction

Aromatic pollutants, including polychlorinated biphenyls (PCBs), polycyclic aromatic hydrocarbons (PAHs), polybrominated diphenyl ethers (PBDEs), and polychlorinated dibenzo-p-dioxins (PCDDs), are a ubiquitous class of environmental contaminants characterized by toxicity, environmental persistence, bioaccumulation, and long-range transport potential, posing substantial threats to ecosystem integrity and human health [1]. These threats are tightly linked to their environmental partitioning behaviors, which are quantified by the *n*-octanol–air partition coefficient (*K*_OA_)—defined as the ratio of the concentration of a chemical in the *n*-octanol phase (*C*_O_) to that in the gas phase (*C*_A_) at the state of distribution equilibrium. *N*-octanol exhibits structural similarity to natural organic matrices, as both are composed of weakly polar to nonpolar components characterized by long alkyl chains and associated minor polar functional groups. This structural congruence enables *n*-octanol to serve as a surrogate for environmental organic phases (e.g., soil humus, biological fats, and plant waxes), allowing it to accurately reflect the partitioning behavior of chemicals in real environmental organic matrices [2]. As a crucial parameter describing pollutant partitioning between the atmosphere and environmental organic phases, *K*_OA_ is essential for modeling transport, predicting environmental distribution, and formulating contamination control strategies [3]. Experimental methods for determining *K*_OA_ include the generation column method, fugacity measurement, and solid-phase microextraction (SPME), while theoretical predictions rely on fragment constants, QSAR models, and solvation free energy methods, collectively supporting environmental and risk studies. Notably, substances with high *K*_OA_ values exhibit strong affinity for organic-rich compartments like soil organic matter, plant waxes, or biological lipids, limiting long-range atmospheric transport and promoting accumulation in near-surface environments [4].

Driven by π–π interactions, chemicals with an aromatic structure could form dimers, trimers, and even polymers in solutions [5,6,7]. During the experimental measurement of the *n*-octanol–air partition coefficient (*K*_OA_) of aromatic pollutants, the concentrations of these chemicals in the *n*-octanol phase are higher than those common in environmental phases, and the probability of molecular encounters and collisions is relatively higher in a typical experimental setting [8,9]. Therefore, it is easier for aromatic chemicals to form aggregates in the *n*-octanol phase. However, the aggregation behavior and the equilibrium characteristics of typical aromatic pollutants in the *n*-octanol phase are not clear. Notably, current approaches for determining *K*_OA_ via experimental measurements or predicting it through theoretical calculations have not considered the impact of such aggregation behavior on *K*_OA_ values [10,11]. It has been reported that the formation of dimers of polychlorinated biphenyls (PCBs) could have a great influence on their apparent *K*_OA_ values [12], with similar findings for polycyclic aromatic hydrocarbons (PAHs), polybrominated diphenyl ethers (PBDEs), polychlorinated naphthalenes (PCNs), and polychlorinated dibenzo-p-dioxins (PCDDs) from theoretical computation [13].

With the improvement of computer performance, molecular dynamics (MD) simulation has become an effective method to investigate the aggregation behavior of substances at the molecular level [14,15,16]. Therefore, MD simulation could be used to investigate the dynamic aggregation behavior and microscopic morphology of aromatic chemicals in the *n*-octanol phase, which are difficult to determine in macroscopic experiments. In order to further analyze these phenomena from a microscopic viewpoint at the molecular level, MD simulation was adopted in the present study to investigate the aggregation process and the equilibrium characteristics of typical aromatic pollutants, including PCB-4, phenanthrene, PBDE-28, PCN-5, and PCDD-1 in the *n*-octanol phase. Furthermore, effects of concentration on the aggregation behavior were investigated in order to analyze the influence of aggregate formation on *K*_OA_. This study could deepen the understanding of the microscale mechanism of environmental partition behavior of aromatic pollutants and improve the theoretical prediction of relevant partition coefficients.

## 2. Materials and Methods

### 2.1. Datasets

In this study, molecular dynamics simulation was employed to investigate the aggregation behavior of typical aromatic pollutants in the *n*-octanol phase under their saturated concentrations. The selected pollutants focused on PCB-4, phenanthrene, PBDE-28, PCN-5, and PCDD-1, which exhibited significant differences in solubility in *n*-octanol. Limited by the availability of experimental values for physicochemical properties, the apparent saturated concentrations in the *n*-octanol phase (*S*_O_) of these typical aromatic pollutants were estimated from the experimentally determined saturated water solubility (*S*_W_) and *n*-octanol–water partition coefficients (*K*_OW_) [17,18,19,20,21,22,23].

(1) $$K_{\mathrm{OW}}=\frac{C_{O}}{C_{W}}=\frac{S_{O}}{S_{W}}$$

where *C*_O_ and *C*_W_ are the concentrations of a chemical in the *n*-octanol phase and the water phase at equilibrium, respectively. In the MD simulation, the number of *n*-octanol molecules was set to 1000, and then the number of molecules of these aromatic pollutants was calculated based on the corresponding estimated *S*_O_. Selected physicochemical properties of these aromatic pollutants and maximal molecule numbers dissolved in the *n*-octanol systems are listed in Table 1.

To systematically evaluate the accuracy of the corrected *K*_OA_ values of aromatic pollutants after correction for aggregation behavior, this study collected experimentally measured log *K*_OA_ values, which are listed in Table 2 [24,25,26,27,28,29,30]. These experimental values covered typical organic pollutants from low to high molecular weights and with different functional group types to ensure the representativeness and coverage of the data. For compounds with multiple reported experimental log *K*_OA_ values from different sources, the average value was calculated and used as the representative experimental data for subsequent comparison. By quantifying the degree of deviation between theoretically corrected log *K*_OA_ values and these experimental data, this study aims to verify the practical effectiveness of the aggregation-effect correction model in improving log *K*_OA_ prediction accuracy. Ultimately, it seeks to provide a scientific basis for the selection of fundamental parameters in subsequent environmental fate simulations and risk assessments of organic compounds.

### 2.2. Molecular Dynamics Simulation

Gromacs 2020.2 software [31] and the GAFF force field [32] were used for the MD simulation. Initial structure files of the target chemicals were obtained online from the PubChem database, topology files with force field parameters were generated using the ACPYPE 2017.1.17 software [33], and the simulation system of typical aromatic pollutants in *n*-octanol solutions was constructed using Gromacs. The steepest descent method was adopted to conduct the initial energy minimization in order to eliminate unreasonable molecular overlap or crossover. After relaxation, the conjugate gradient method was used to minimize the energy once again, so that the structure of the system and the distance between atoms and the configuration were reasonable.

After energy minimization, the system was pre-balanced for 1000 ps in the canonical ensemble (NVT), followed by 1000 ps in the constant-pressure, constant-temperature ensemble (NPT). The NVT ensemble was used to reduce the pressure of the simulation box and heat the system to the set temperature. NPT ensemble was used to adjust the pressure of the model system to reach the convergence of the density. Then, MD simulation was performed for 50 ns after the system reached equilibrium. The integral algorithm used in the MD simulation was the leap-frog method [34] with a step size of 1 fs. The long-range electrostatic interaction was calculated using the cut-off method [35], in which the cut-off distance for the non-bonded interactions was set to 1.2 nm. The pressure was controlled to 1 atm by using isotropic Parrinello–Rahman pressure coupling [36]. Temperature coupling was achieved using the velocity rescaling heat bath method [37], and the temperature was controlled at 298 K. The linear constraint solver (LINCS) algorithm was used to restrict all chemical bonds [38]. Three-dimensional periodic boundary conditions were used to remove the size effects.

The system structure and thermodynamic properties were detected to determine whether the final equilibrium had been reached. Trajectory files containing the positions and speeds of all particles in the system were obtained. Then, useful thermodynamic and statistical information was calculated from the trajectory files. In addition, the visual molecular dynamics software (VMD) 1.9.3 [39] was used to depict trajectory snapshot graphs, display the particle movement trajectory, and analyze the particle movement in the simulation system. A dimer with the face-to-face, offset face-to-face, or edge-to-face conformation was recognized when the centroid distance of two molecules was shorter than 3.8, 3.9, or 5.0 Å, respectively [40,41].

### 2.3. Calculation of log K_OA_ and Solvation Free Energy of Aromatic Pollutants

According to fundamental thermodynamic principles, the logarithm of the *n*-octanol–air partition coefficient (log *K*_OA_) can be derived from the Gibbs free energy of solvation from air to *n*-octanol (Δ*G*_OA_, herein referred to as solvation free energy).

(2) $$log\;K_{OA}=-\frac{\Delta G_{OA}}{2.303\;RT}$$

This quantitative relationship is specifically governed by thermodynamic equations involving the gas constant (R = 8.314 J·mol^−1^·K^−1^) and absolute temperature (T, in Kelvin). Such a thermodynamic correlation facilitates the development of an efficient approach for directly estimating log *K*_OA_ values using Δ*G*_OA_, reducing reliance on labor-intensive experiments—particularly as quantum chemistry enables high-precision Δ*G*_OA_ calculations. For solvation free energy estimation, this study employed the Solvation Model Density (SMD) model developed by Marenich et al. [42], selected for its superior performance in capturing solute–solvent interactions across diverse chemical systems and its proven accuracy in thermodynamic property calculations.

Molecular structures of target aromatic pollutant congeners and dimers were constructed using CS ChemDraw Ultra (Version 14.0, Cambridge Scientific Computing, Inc., Cambridge, UK). The calculation of Δ*G*_OA_ based on the SMD involved three sequential steps: geometry optimization, frequency calculation, and single-point energy calculation. All computational procedures were performed using the Gaussian 09-E01 software package [43]. Frequency calculations were conducted to verify that the optimized molecular geometries correspond to true minima on the potential energy surface, ensuring the reliability of subsequent energy calculations. Single-point energies in both the gas phase and *n*-octanol phase were computed using the SMD model at the HF/MIDI!6D theoretical level—a combination previously validated as optimal for solvation free energy calculations. Finally, log *K*_OA_ values were determined from the computed Δ*G*_OA_ values using Equation (2).

### 2.4. Study on Effects of the Concentration on the Aggregation Behavior

The effect of the concentration on the aggregation behavior of aromatic pollutants was explored based on six concentrations. These concentrations were the saturation concentration, one-half, one-quarter, one-eighth, one-sixteenth, and one-thirty-second of the saturation concentration in the *n*-octanol phase. Corresponding molecular numbers of aromatic pollutants and *n*-octanol at different concentrations are listed in Table S1. The molecular conformations at different concentrations after 50 ns of MD simulation were captured to analyze the aggregation behavior of these typical aromatic pollutants in the *n*-octanol phase.

The effect of the concentration was further analyzed by comparing the aggregation behavior of these aromatic pollutants at the same concentration level. The concentration was set to be their lowest apparent saturated concentration (9.67 × 10^−2^ mol/L) in the *n*-octanol phase, corresponding to 61 aromatic molecules dissolved in 4000 *n*-octanol molecules. After 50 ns of MD simulation, the molecular conformations of different aromatic pollutants at the same concentration were captured and analyzed.

## 3. Results and Discussion

### 3.1. The Aggregation Processes of Typical Aromatic Pollutants in the n-Octanol Phase

Changes in the potential energy of *n*-octanol systems saturated with typical aromatic pollutants during the MD simulation were presented in Figure S1. It can be seen that the initial potential energies were about 15,000 to 16,000 kcal/mol, and they quickly decreased in the initial 30 ps. Thereafter, the downward trend gradually slowed down. After 50 ps, the potential energies of these systems tended to be stable, and finally fluctuated around 10,762, 10,706, 10,062, 10,361, and 10,521 kcal/mol, respectively, within small ranges. The total energy of the systems presented a similar trend as the potential energy, and finally fluctuated around 29,821, 30,056, 29,160, 29,213, and 29,734 kcal/mol, respectively (Figure S2). These results indicated that the systems almost reached equilibrium after 50 ps of MD simulation.

In order to analyze the aggregation process of the typical aromatic pollutants in the *n*-octanol phase, molecular conformations at different simulation times were captured, as shown in Figures S3–S7: PCB-4 (Figure S3), phenanthrene (Figure S4), PBDE-28 (Figure S5), PCN-5 (Figure S6), and PCDD-1 (Figure S7). The *n*-octanol solvent molecules were concealed in these figures, while the molecules of aromatic pollutants were retained in order to show their aggregation behaviors clearly. It can be seen that the molecular aggregation processes were similar over time. At the beginning, molecules of the aromatic pollutants were randomly distributed in the solution inside the box. At 5 ps, some molecules were sufficiently close to each other to form dimers. During 5–30 ps, the molecular conformation changed greatly, and the number of dimers increased gradually. After 30 ps, the aggregation of these aromatic molecules in the systems gradually slowed down. At 50 ps, the systems were relatively stable with three dimers formed for PCB-4, five dimers for phenanthrene, four dimers for PBDE-28, three dimers for PCN-5, and seven dimers and one trimer for PCDD-1. Together with Figures S1 and S2, it can be confirmed that the equilibrium was quickly reached following significant changes in the molecular conformation as well as the plunge of potential energy and total energy of the systems.

Changes in aggregate percentages of these aromatic pollutants were summarized in Table 3. It can be seen that the proportion of dimers gradually increased and the proportion of single molecules gradually decreased with increasing simulation time. At 50 ps, the proportion of dimers presented the order of PCDD-1 > PCN-5 > PBDE-28 > Phenanthrene > PCB-4. PCDD-1 had the highest proportion of aggregates among these five typical aromatic pollutants. In addition to dimers, PCDD-1 also formed a trimer at 50 ps.

The molecular aggregation process of these aromatic pollutants could be divided into three stages: random motion stage, aggregation stage, and adjustment and equilibrium stage, as described by [44]. During the random motion stage, the aromatic molecules collided with surrounding molecules and moved randomly. When two aromatic molecules got closer to each other during the random collision, the molecules could be attracted by intermolecular interactions, such as hydrogen bond interactions and π–π interactions. It was found that only 1~2 hydrogen bonds could be formed in the simulation process. Therefore, π–π interactions should be the main driving force for the aggregation of aromatic molecules. With the decrease of molecular distance, the π–π interactions gradually increased. However, the molecules could not be infinitely close to each other due to the stereo-hindrance effect and electrostatic repulsion. During this aggregation stage, aggregates were formed and the energy of the systems decreased greatly. After this, the systems entered the adjustment and equilibrium stage. At this stage, the aromatic molecules in the aggregates continuously adjusted their positions to stabilize the structure and the final dimers existed mostly in the form of edge-to-face stacking and offset face-to-face stacking.

### 3.2. Aggregation Characteristics of Typical Aromatic Pollutants in the n-Octanol Phase at Equilibrium

The simulation time was further extended to 50 ns to investigate the aggregation behavior of these aromatic pollutants in the *n*-octanol phase. Firstly, two molecules in a system were randomly selected to analyze the change of their centroid distance (Figure S8). The changes in the molecular centroid distance with the simulation time are shown in Figure S9. It can be seen that the molecular centroid distances changed irregularly with the simulation time. Sometimes, the molecular centroid distance was less than the corresponding cut-off distance of dimers, suggesting that dimers had been formed and separated for many times during the MD simulation. Therefore, a dynamic equilibrium of aggregation and segregation had established in the systems.

In order to directly reflect the dynamic equilibrium process, changes in the molecular conformations at different simulation times were captured and shown in Figures S10–S14. It could be seen that there were always three dimers for PCB-4, although they were present in different places of the simulation system within 50 ns. This also suggested that there existed a dynamic equilibrium of aggregation and segregation for PCB-4 dimers and the number of dimers was always constant in the system. This was the case for phenanthrene, PBDE-28, PCN-5, and PCDD-1, though different numbers of dimers were formed. Based on Figures S10–S14, the aggregate percentages of these aromatic pollutants were summarized and listed in Table 4. It showed that the aggregate percentages of these aromatic pollutants remained almost constant in the equilibrium state, except for the systems of PCN-5 and PCDD-1 at 20 ns and 10 ns respectively. The aggregate percentages also followed the order of PCDD-1 > PCN-5 > PBDE-28 > phenanthrene > PCB-4.

It is known that π–π interactions are the main driving force for the aggregation of aromatic molecules. Therefore, π–π interactions of PCDD-1, PCN-5, PBDE-28, phenanthrene, and PCB-4 were calculated and determined to be −11.54, −11.10, −10.98, −10.37, and −9.77 kcal/mol, respectively. It was found that the values gradually increased, indicating that the corresponding π–π interactions decreased gradually. It is believed that the stronger the π–π interaction, the more aggregates are formed. Therefore, the aggregation degree of these aromatic pollutants decreased in the order of PCDD-1, PCN-5, PBDE-28, phenanthrene, and PCB-4, as expected.

Aromatic pollutants can form aggregates in the *n*-octanol phase, while they usually exist as individual molecules in the gas phase. It has been reported that the log *K*_OA_ of organic chemicals could be predicted from the solvation free energy from the gas phase to the *n*-octanol phase (Δ*G*_OA_) [11]. The aggregates formed in the *n*-octanol phase could affect the Δ*G*_OA_ of aromatic pollutants and consequently affect their *K*_OA_ values. Based on aggregate percentages of these aromatic pollutants at equilibrium and Δ*G*_OA_ calculated for different aggregates, their log *K*_OA_ values were estimated (Table S2). It can be seen from the table that the estimated log *K*_OA_ values were close to the experimental ones. The deviation between experimental and estimated log *K*_OA_, could be related to the different concentrations considered. For PCDD-1, there is a trend to form more polymers, and therefore the experimental value was slightly higher than the estimated value.

### 3.3. Effects of the Concentration on the Aggregation Behavior and K_OA_ of Typical Aromatic Pollutants

**Figure 1:**
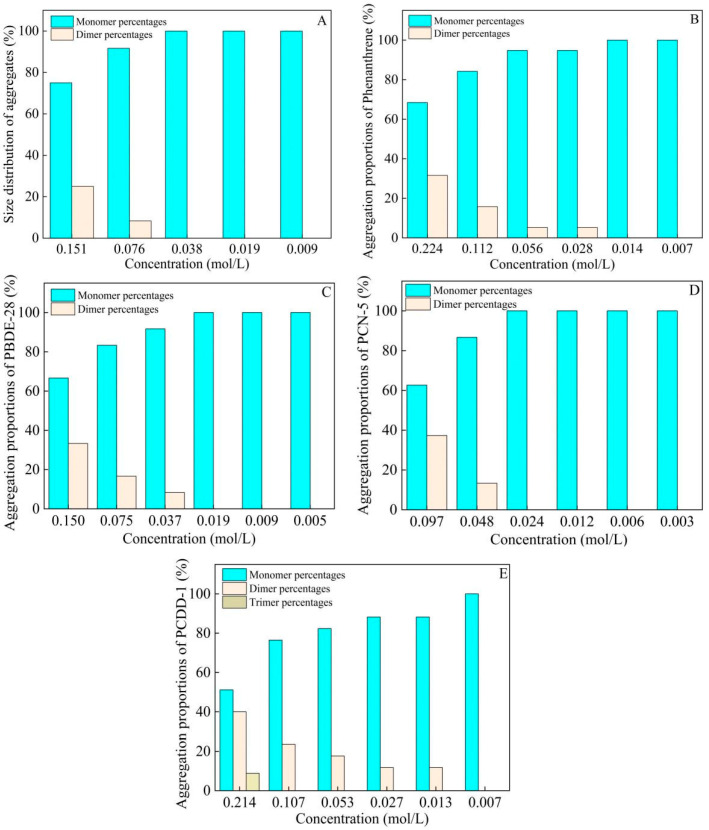
Aggregation proportions of PCB-4 (**A**), phenanthrene (**B**), PBDE-28 (**C**), PCN-5 (**D**), and PCDD-1 (**E**) formed in the *n*-octanol phase at different concentrations under equilibrium state.

The molecular conformations of these typical aromatic pollutants in the *n*-octanol phase at different concentrations are shown in Figures S15–S19. It can be seen that the concentration greatly affected the aggregation behavior of these aromatic pollutants. The proportion of aggregates decreased with decreasing concentrations of aromatic pollutants in the *n*-octanol phase. When the concentrations of PCB-4, phenanthrene, PBDE-28, PCN-5, and PCDD-1 decreased to one-fourth, one-sixteenth, one-eighth, one-fourth, and one-thirty-second of the saturated concentration, respectively, these aromatic pollutants existed completely in monomeric form. The aggregate proportions of aromatic pollutants formed in the *n*-octanol phase at different concentrations are summarized in Figure 1. When the concentrations of PCB-4, phenanthrene, PBDE-28, PCN-5, and PCDD-1 in the *n*-octanol phase decreased to 0.038, 0.014, 0.019, 0.024, and 0.007 mol/L, respectively, the aromatic pollutants completely existed in monomeric form, without any dimers or trimers.

The MD simulations were used to estimate the monomer percentages and aggregate percentages of these aromatic pollutants, and, subsequently, their apparent log *K*_OA_ values were estimated based on these percentages and corresponding Δ*G*_OA_ values, calculated following the method reported by Li et al. [11] The estimated log *K*_OA_ values are listed in Tables S3–S7. With the decrease in concentration, the estimated log *K*_OA_ values gradually decreased, indicating that the *K*_OA_ values of these aromatic pollutants are not constants, but change with concentration. It can be seen that different numbers of aggregates formed in the *n*-octanol phase at different concentrations. As the hydrophobicity of dimers and polymers is higher than the hydrophobicity of monomers, the presence of dimers and polymers will increase the apparent *K*_OA_ values. The higher the concentration, the more aggregates formed, and consequently the higher the apparent log *K*_OA_ value.

When the concentrations of these aromatic pollutants in the *n*-octanol phase were kept the same as 9.67 × 10^−2^ mol/L (61 aromatic molecules: 4000 *n*-octanol molecules), the aggregation behavior of different aromatic pollutants was analyzed, and the results are shown in Figure S20. At the equilibrium state, there were four dimers formed for PCB-4, four dimers for phenanthrene, eight dimers for PBDE-28, 12 dimers for PCN-5, and six dimers for PCDD-1. The percentages of aggregates formed by these aromatic pollutants are summarized in Table 5. The results showed that these aromatic pollutants all had aggregates in the *n*-octanol phase at this concentration. However, the proportion of aggregates formed was in the order of PCN-5 > PBDE-28 > PCDD-1 > phenanthrene = PCB-4. This order was different from that at the saturation concentrations shown in Table 3. This might be related to different degrees of concentration decrease and different rates of aggregate proportion decline following the decrease in concentrations.

At the concentration of 9.67 × 10^−2^ mol/L, the log *K*_OA_ values of these aromatic pollutants were estimated based on the percentages and corresponding Δ*G*_OA_ values, the results of which are also shown in Table 4. It was interesting to find that the log *K*_OA_ value of PCB-4 estimated at this concentration was closer to the corresponding experimental value than the value estimated at saturation concentrations. This suggests that the concentration of aromatic pollutants affects their aggregation behaviors and, consequently, *K*_OA_ values, and that the experimental log *K*_OA_ values should be measured at unsaturated concentrations. Therefore, the partition behavior of aromatic pollutants in the actual environment could be different from that in the experimental measurement setting, and the effect of the concentration on log *K*_OA_ values should be taken into consideration when the environmental behavior and transport of these aromatic pollutants are investigated.

## 4. Conclusions

Our study demonstrates that aromatic pollutants can form dimers and even trimers in the *n*-octanol phase, with their abundance increasing at higher concentrations. These aggregates exhibit enhanced hydrophobicity, thereby influencing the partitioning behavior of aromatic pollutants. Notably, aggregation is markedly more pronounced in liquid phases (such as *n*-octanol or water) compared to the gas phase, where monomers predominate. Consequently, aggregation exerts a stronger effect on the *K*_OA_ than on *K*_OW_. Experimentally derived log *K*_OA_ values were found to decrease with decreasing concentration, reflecting aggregate dissociation. Since conventional measurements employ high concentrations, the resulting log *K*_OA_ values likely overestimate both aggregation tendency and partition coefficients under environmental conditions. Thus, ambient concentration levels must be considered when applying log *K*_OA_ values to predict the environmental distribution and fate of aromatic pollutants. Furthermore, π–π interactions were identified as the key driving force for aggregation, with variations among pollutants explaining differences in their *K*_OA_ values. This study highlights the critical roles of concentration-dependent aggregation and π–π interactions in modulating the partitioning behavior of aromatic pollutants, with significant implications for accurate risk assessment and environmental modeling.

## Figures and Tables

**Table 1:** Selected physicochemical properties of typical aromatic pollutants and maximal molecule numbers dissolved in 1000 *n*-octanol molecules.

Chemicals	*S*_W_ (mol/L)	log *K*_OW_	*S*_O_ (mol/L)	*n* (Chemical):*n* (*n*-Octanol)
PCB-4	1.91 × 10^−6^	4.90	1.51 × 10^−1^	24:1000
Phenanthrene	6.03 × 10^−6^	4.57	2.24 × 10^−1^	35:1000
PBDE-28	1.72 × 10^−7^	5.94	1.50 × 10^−1^	24:1000
PCN-5	1.60 × 10^−6^	4.78	9.67 × 10^−2^	15:1000
PCDD-1	1.91 × 10^−6^	5.05	2.14 × 10^−1^	34:1000

**Table 2:** Experimental and average log *K*_OA_ values of typical aromatic pollutants.

Chemicals	log *K*_OA_		Reference
	Experimental Values	Average Values	
PCB-4	7.18	7.18	[24]
Phenanthrene	7.45	7.65	[25]
	7.57		[26]
	7.88		[27]
	7.68		[28]
PBDE-28	9.50	9.50	[29]
PCN-5	6.93	6.93	[26]
PCDD-1	7.86	7.86	[30]

**Table 3:** Changes in aggregate percentages of typical aromatic pollutants in the aggregation process.

Aggregate Form	Time (ps)	Aggregate Percentages (%)				
		PCB-4	Phe	PBDE-28	PCN-5	PCDD-1
Monomer	0	100.0	100.0	100.0	100.0	100.0
	5	91.7	88.6	91.7	100.0	82.4
	10	83.3	88.6	83.3	86.7	82.4
	20	83.3	82.9	83.3	86.7	76.5
	30	75.0	77.1	75.0	60.0	70.6
	50	75.0	71.4	66.7	60.0	50.0
Dimer	0	0	0	0	0	0
	5	8.3	11.4	8.3	0	17.7
	10	16.7	11.4	16.7	13.3	17.7
	20	16.7	17.1	16.7	13.3	23.5
	30	25.0	22.9	25.0	40.0	29.4
	50	25.0	28.6	33.3	40.0	41.2
Trimer	0	0	0	0	0	0
	5	0	0	0	0	0
	10	0	0	0	0	0
	20	0	0	0	0	0
	30	0	0	0	0	0
	50	0	0	0	0	8.8

**Table 4:** Change of aggregate percentages of typical aromatic pollutants at different simulation times under equilibrium state.

Aggregate Form	Time (ns)	Aggregate Percentages (%)				
		PCB-4	Phe	PBDE-28	PCN-5	PCDD-1
Monomer	0	75.0	71.4	66.7	60.0	50.0
	10	75.0	71.4	66.7	60.0	55.9
	20	75.0	71.4	66.7	73.3	50.0
	30	75.0	71.4	66.7	60.0	50.0
	40	75.0	71.4	66.7	60.0	50.0
	50	75.0	71.4	66.7	60.0	50.0
Dimer	0	25.0	28.6	33.3	40.0	41.2
	10	25.0	28.6	33.3	40.0	35.3
	20	25.0	28.6	33.3	26.7	41.2
	30	25.0	28.6	33.3	40.0	41.2
	40	25.0	28.6	33.3	40.0	41.2
	50	25.0	28.6	33.3	40.0	41.2
Trimer	0	0	0	0	0	8.8
	10	0	0	0	0	8.8
	20	0	0	0	0	8.8
	30	0	0	0	0	8.8
	40	0	0	0	0	8.8
	50	0	0	0	0	8.8

**Table 5:** Aggregate percentages in the *n*-octanol phase at 9.67 × 10^−2^ mol/L and estimated log *K*_OA_ values of typical aromatic pollutants.

Chemicals	Monomer Percentages (%)	Dimer Percentages (%)	Trimer Percentages (%)	Estimated log *K*_OA_ Values
PCB-4	86.9	13.1	0	7.24
Phe	86.9	13.1	0	7.17
PBDE-28	73.8	26.2	0	9.19
PCN-5	60.0	40.0	0	6.85
PCDD-1	80.3	19.7	0	5.72

## Data Availability

The original contributions presented in this study are included in the article. Further inquiries can be directed to the corresponding author.

## Supplementary Materials

The following supporting information can be downloaded at: https://www.mdpi.com/article/10.3390/toxics13090721/s1, Figure S1: Changes of the potential energy of *n*-octanol systems saturated with PCB-4 (A), Phenanthrene (B), PBDE-28 (C), PCN-5 (D) and PCDD-1 (E) during the MD simulation; Figure S2: Changes of the total energy of *n*-octanol systems saturated with PCB-4 (A), Phenanthrene (B), PBDE-28 (C), PCN-5 (D) and PCDD-1 (E) during the MD simulation; Figure S3: The aggregation process of PCB-4 molecules in the *n*-octanol phase (Red circles marked for dimers); Figure S4: The aggregation process of Phenanthrene molecules in the *n*-octanol phase (Red circles marked for dimers); Figure S5: The aggregation process of PBDE-28 molecules in the *n*-octanol phase (Red circles marked for dimers); Figure S6: The aggregation process of PCN-5 molecules in the *n*-octanol phase (Red circles marked for dimers); Figure S7: The aggregation process of PCDD-1 molecules in the *n*-octanol phase (Red circles marked for dimers and yellow circle for trimer); Figure S8: Initial centroid distances of PCB-4 (A), Phenanthrene (B), PBDE-28 (C), PCN-5 (D) and PCDD-1 (E); Figure S9: Changes of molecular centroid distance of PCB-4 (A), Phenanthrene (B), PBDE-28 (C), PCN-5 (D) and PCDD-1 (E) with the simulation time; Figure S10: Changes of molecular conformation of PCB-4 at different simulation time (Red circles marked for dimers); Figure S11: Changes of molecular conformation of Phenanthrene at different simulation time (Red circles marked for dimers); Figure S12: Changes of molecular conformation of PBDE-28 at different simulation time (Red circles marked for dimers); Figure S13: Changes of molecular conformation of PCN-5 at different simulation time (Red circles marked for dimers); Figure S14: Changes of molecular conformation of PCDD-1 at different simulation time (Red circles marked for dimers and yellow circle for trimer); Figure S15: Molecular conformations of PCB-4 in the *n*-octanol phase at different concentrations (Red circles marked for dimers); Figure S16: Molecular conformations of Phenanthrene in the *n*-octanol phase at different concentrations (Red circles marked for dimers); Figure S17: Molecular conformations of PBDE-28 in the *n*-octanol phase at different concentrations (Red circles marked for dimers); Figure S18: Molecular conformations of PCN-5 in the *n*-octanol phase at different concentrations (Red circles marked for dimers); Figure S19: Molecular conformations of PCDD-1 in the *n*-octanol phase at different concentrations (Red circles marked for dimers and yellow circle for trimer); Figure S20: Aggregation behavior of different aromatic pollutants in the *n*-octanol phase at the same concentration of 9.67×10-2 mol/L (Red circles marked for dimers); Table S1: Molecular number of aromatic pollutants and *n*-octanol calculated at different concentrations; Table S2: Experimental and estimated log *K*_OA_ of 5 typical aromatic pollutants based on predicted aggregate percentages and their molecular structures; Table S3: Aggregate percentages, and experimental and estimated log *K*_OA_ values of PCB-4 at different concentrations; Table S4: Aggregate percentages, and experimental and estimated log *K*_OA_ values of Phenanthrene at different concentrations; Table S5: Aggregate percentages, and experimental and estimated log *K*_OA_ values of PBDE-28 at different concentrations; Table S6: Aggregate percentages, and experimental and estimated log *K*_OA_ values PCN-5 at different concentrations; Table S7: Aggregate percentages, and experimental and estimated log KOA values of PCDD-1 at different concentrations.
