# Supplementary material for: Molecular Dynamics Simulation of the Aggregation Behavior of Typical Aromatic Pollutants and Its Influence on the n-Octanol–Air Partition Coefficient
Source: Toxics. 2025 Aug 28;13(9):721. doi: 10.3390/toxics13090721 (PMC12473948; doi:10.3390/toxics13090721)
Supplement: Supplementary file 1 [file toxics-13-00721-s001.zip › toxics-3802187-supplementary.pdf]

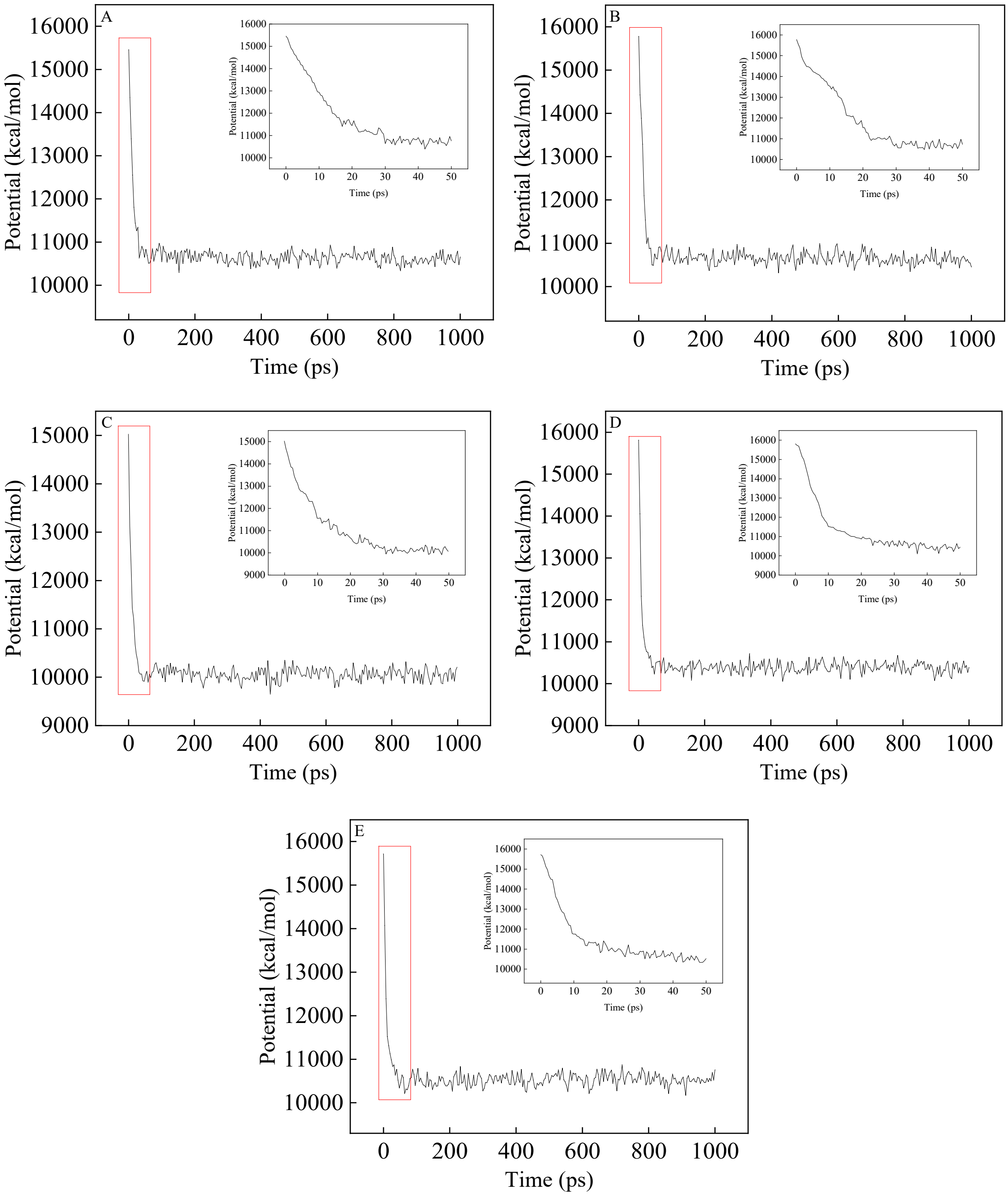

Figure S1. Changes of the potential energy of *n*-octanol systems saturated with PCB-4 (A), Phenanthrene (B), PBDE-28 (C), PCN-5 (D) and PCDD-1 (E) during the MD simulation.

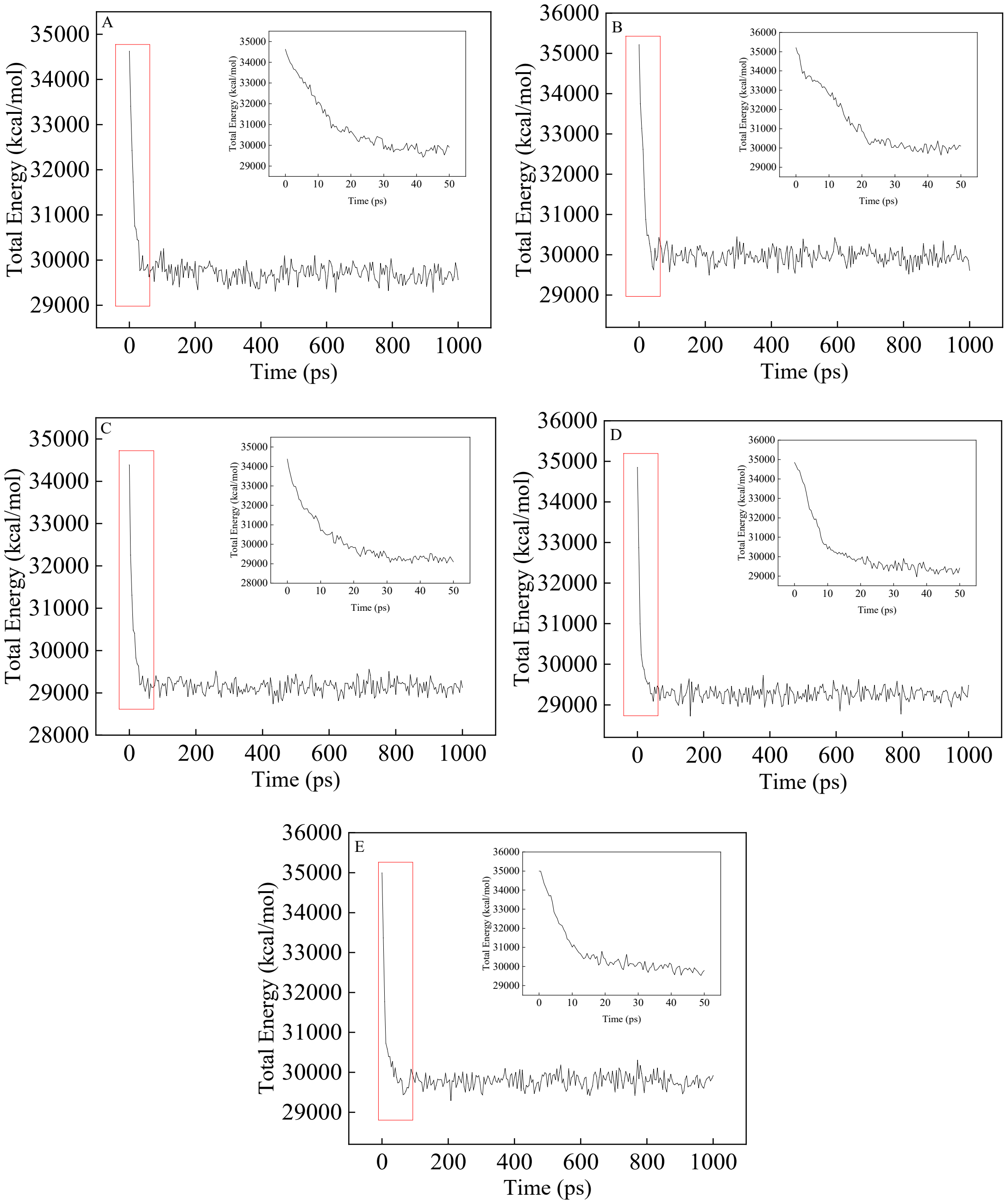

Figure S2. Changes of the total energy of *n*-octanol systems saturated with PCB-4 (A), Phenanthrene (B), PBDE-28 (C), PCN-5 (D) and PCDD-1 (E) during the MD simulation.

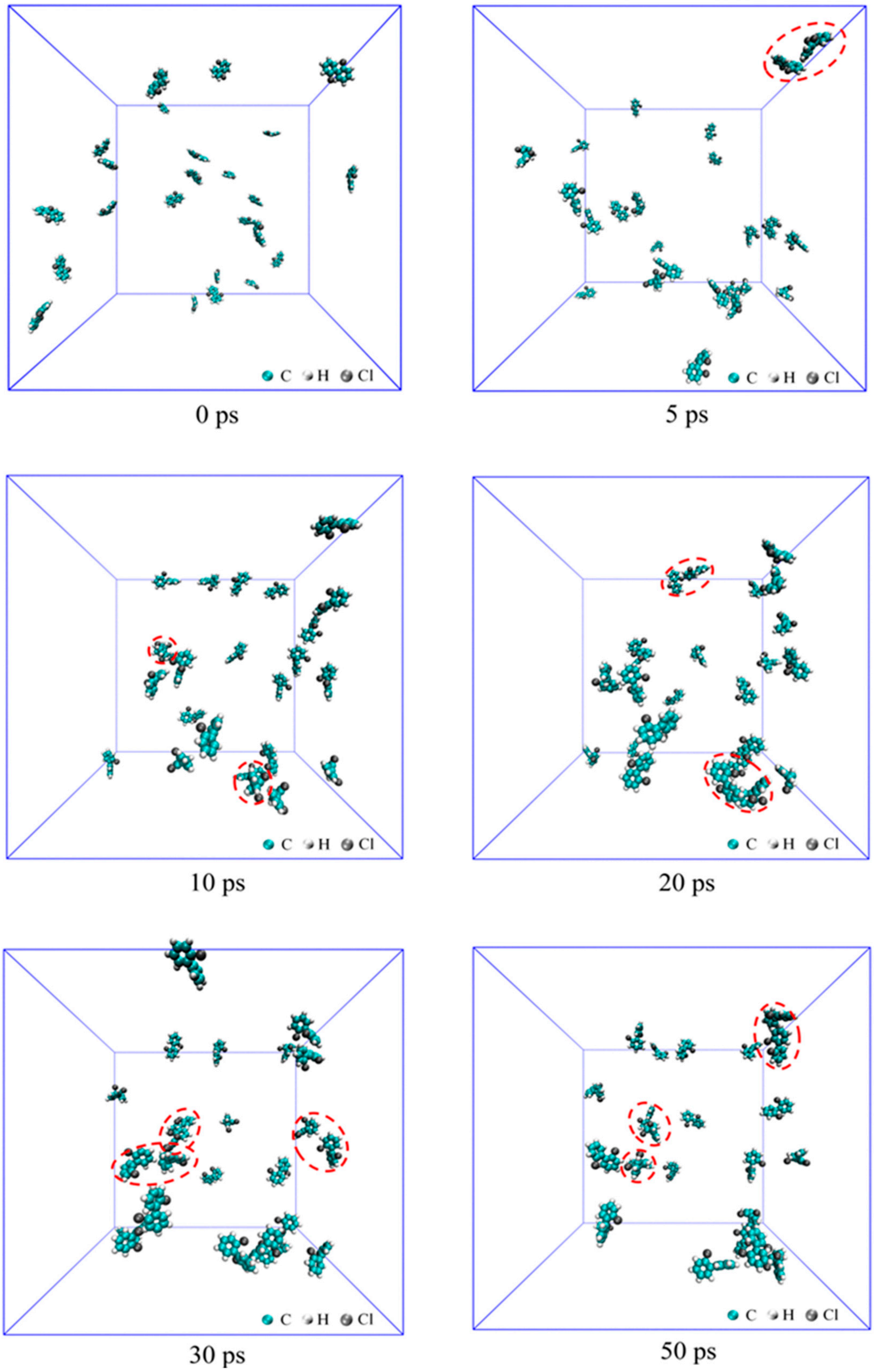

Figure S3. The aggregation process of PCB-4 molecules in the *n*-octanol phase (Red circles marked for dimers).

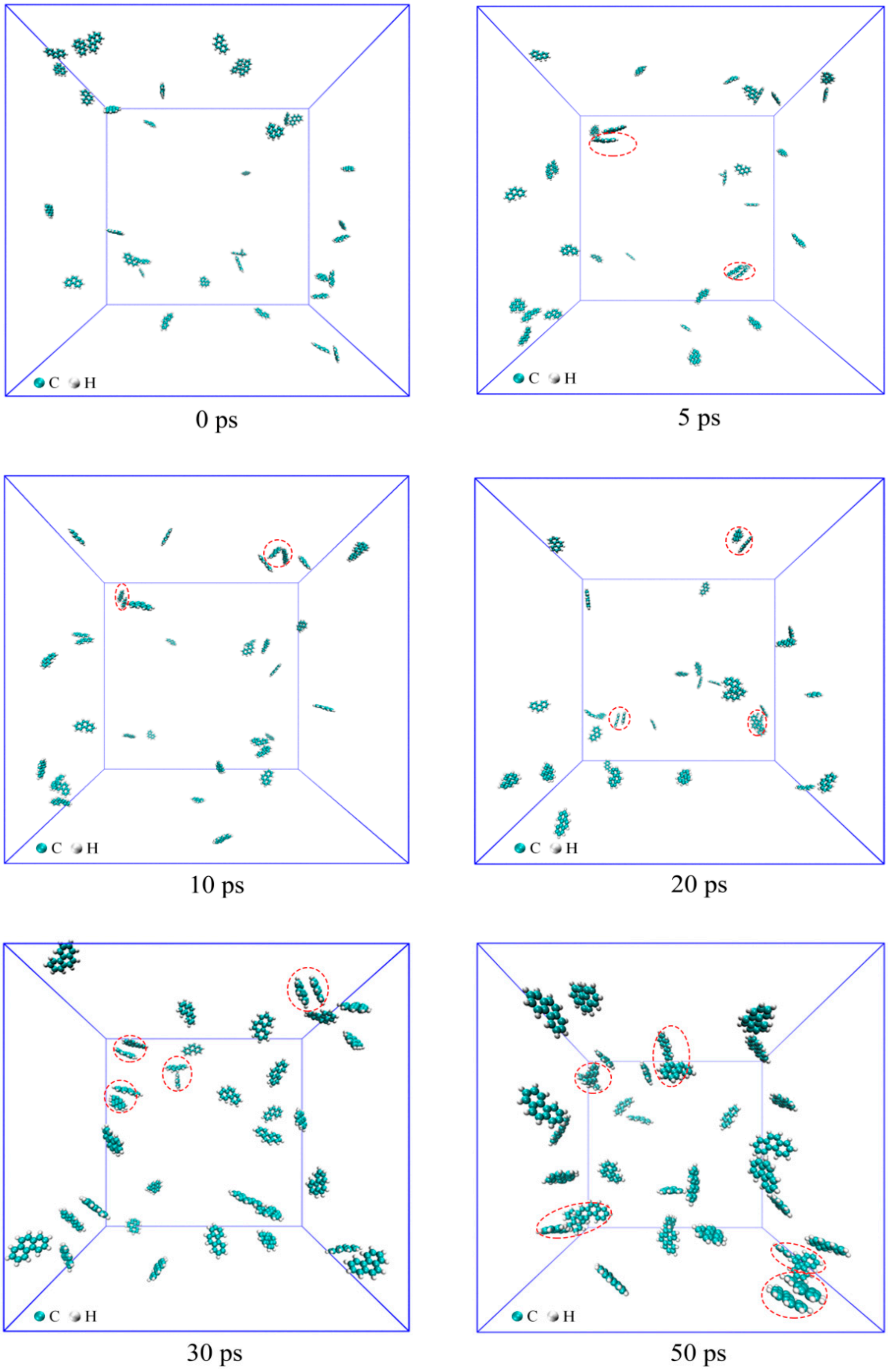

Figure S4. The aggregation process of Phenanthrene molecules in the *n*-octanol phase (Red circles marked for dimers).

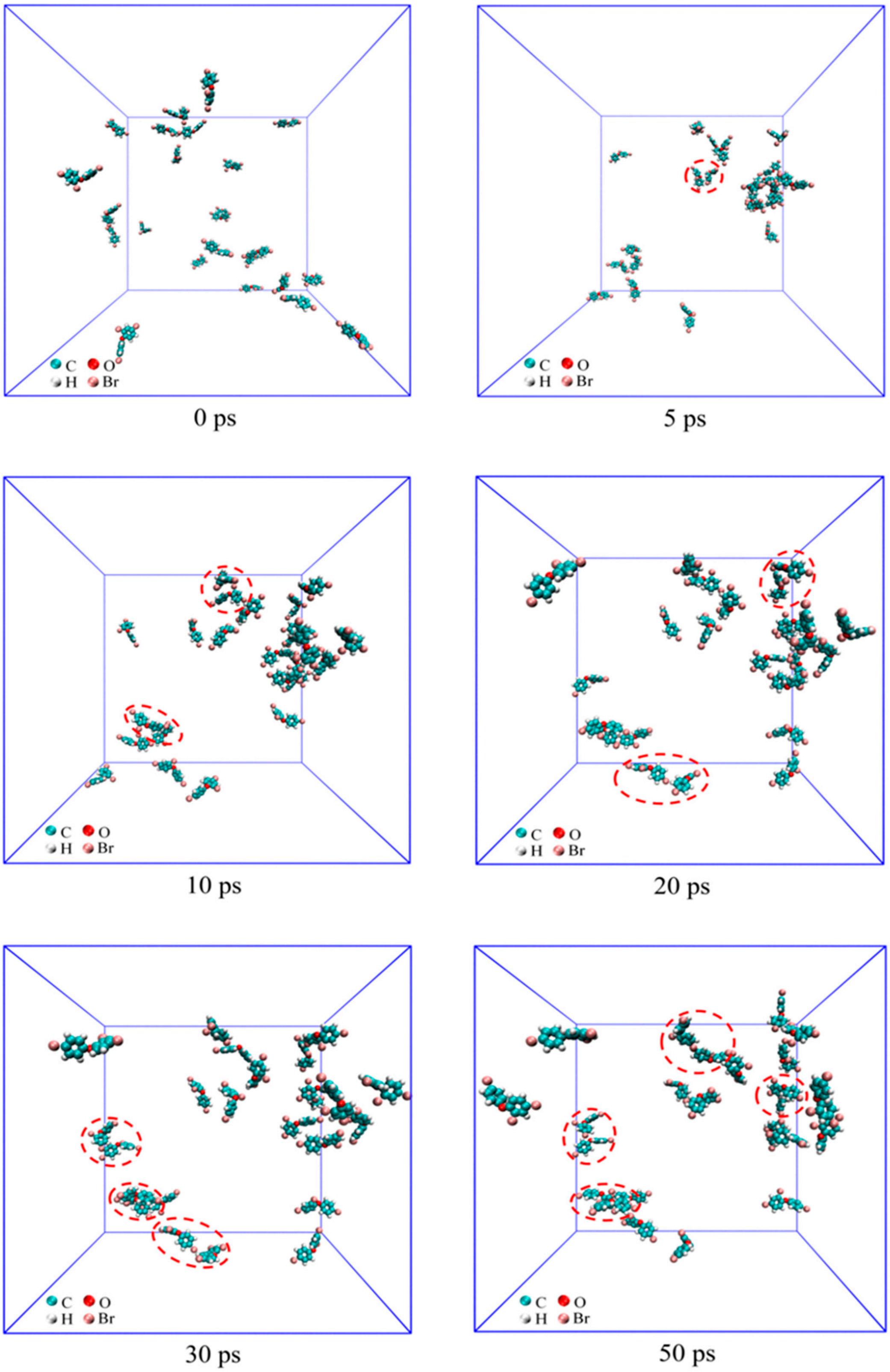

Figure S5. The aggregation process of PBDE-28 molecules in the *n*-octanol phase (Red circles marked for dimers).

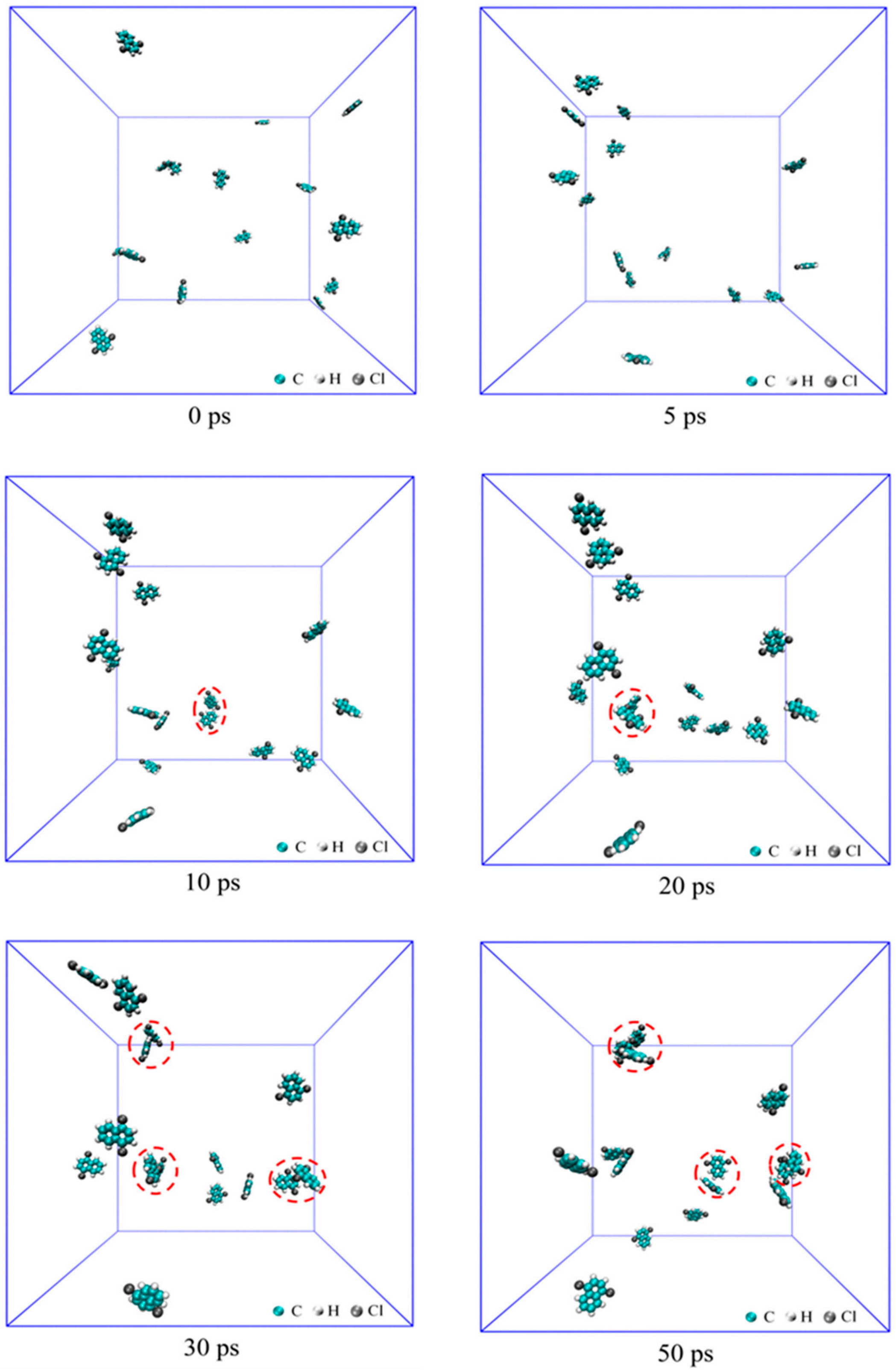

Figure S6. The aggregation process of PCN-5 molecules in the *n*-octanol phase (Red circles marked for dimers).

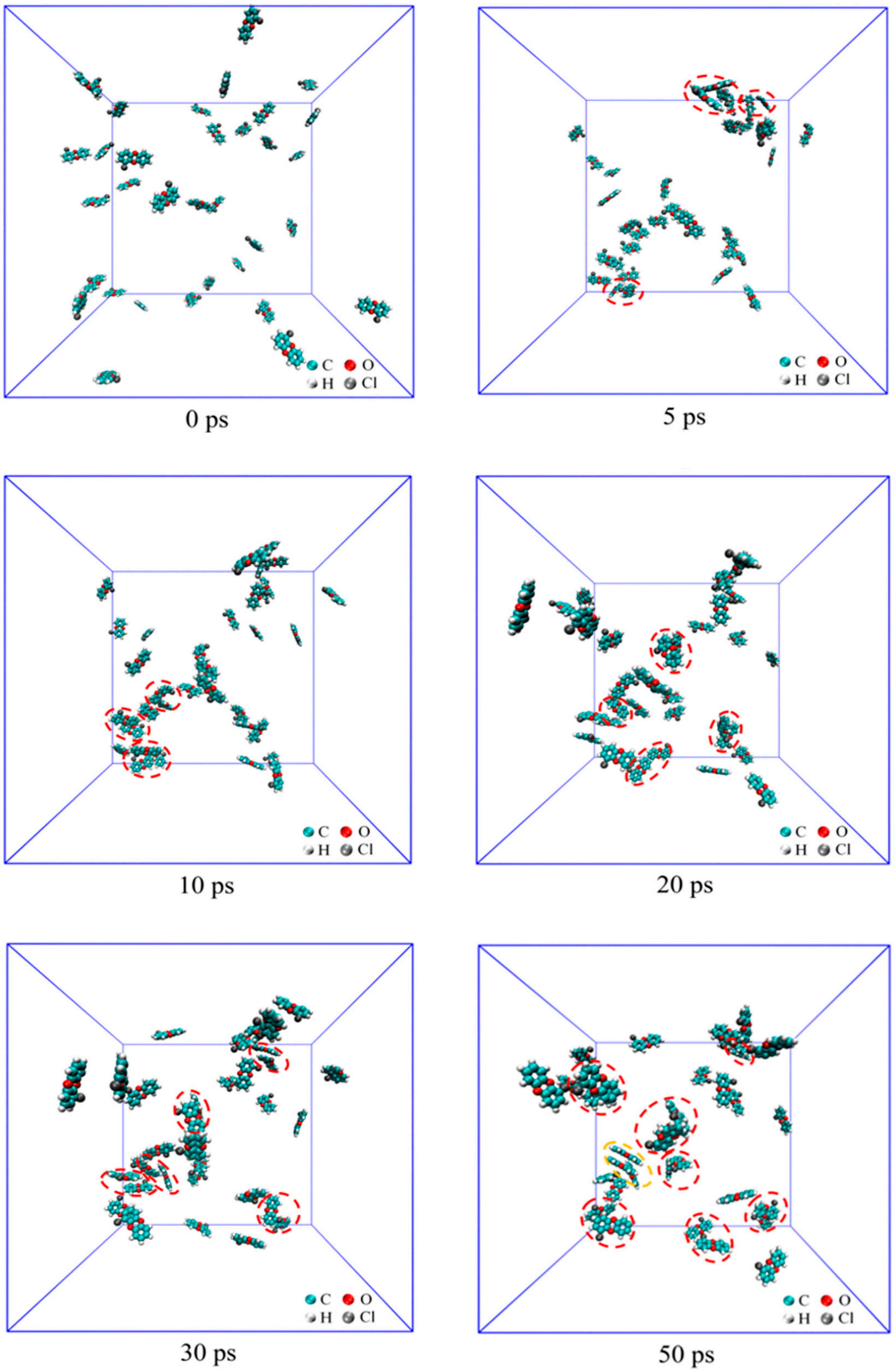

Figure S7. The aggregation process of PCDD-1 molecules in the *n*-octanol phase (Red circles marked for dimers and yellow circle for trimer).

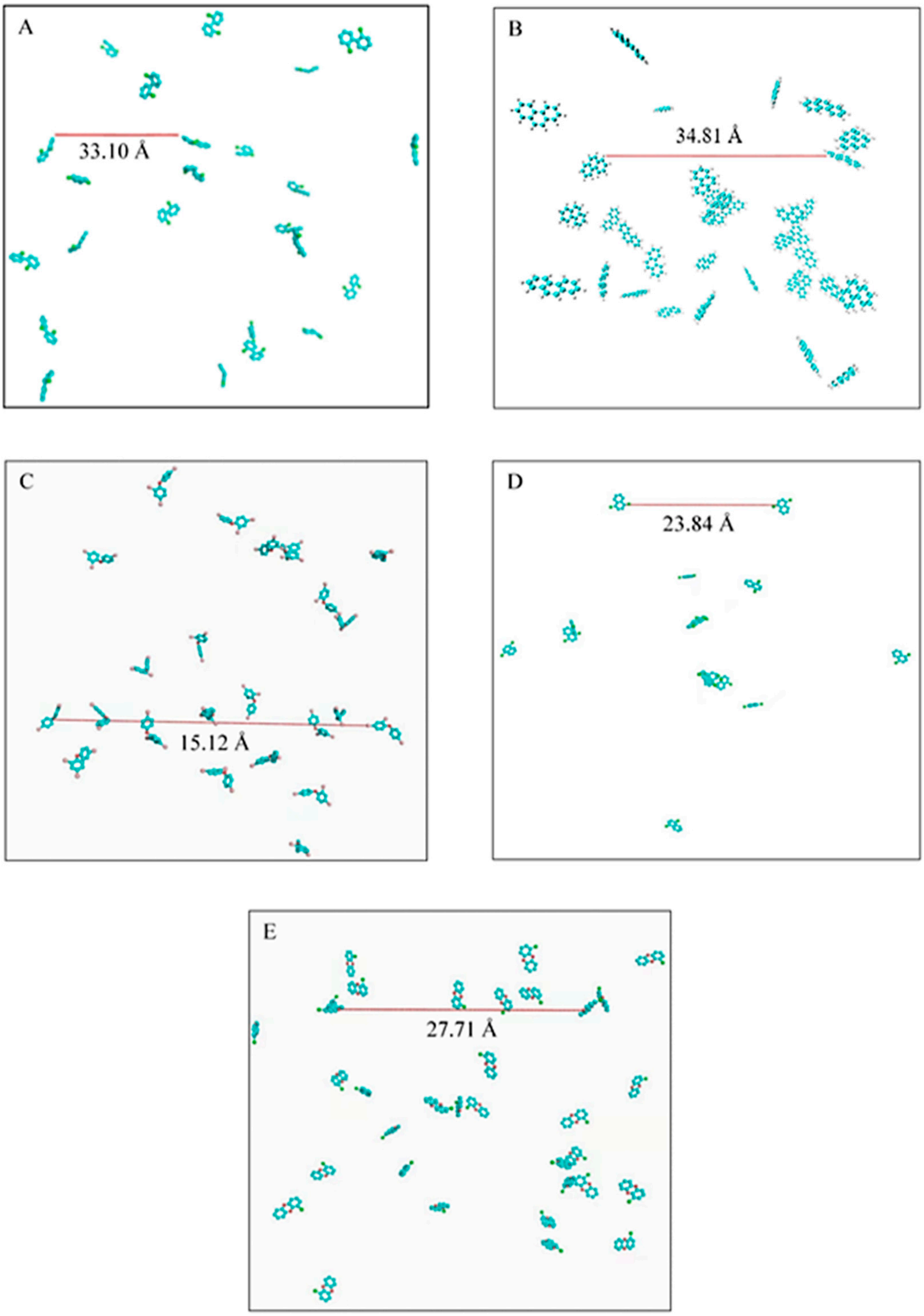

Figure S8. Initial centroid distances of PCB-4 (A), Phenanthrene (B), PBDE-28 (C), PCN-5 (D) and PCDD-1 (E).

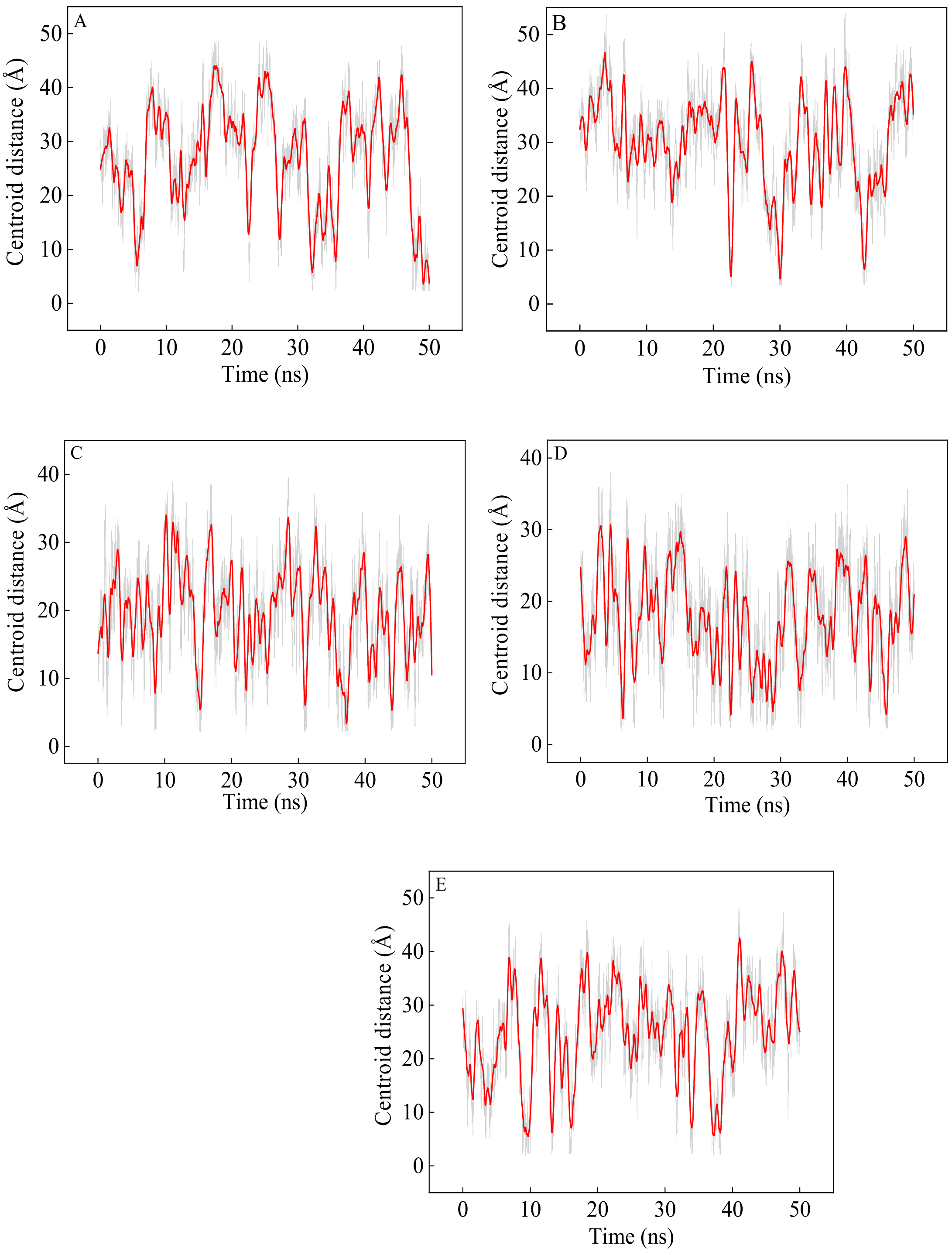

Figure S9. Changes of molecular centroid distance of PCB-4 (A), Phenanthrene (B), PBDE-28 (C), PCN-5 (D) and PCDD-1 (E) with the simulation time.

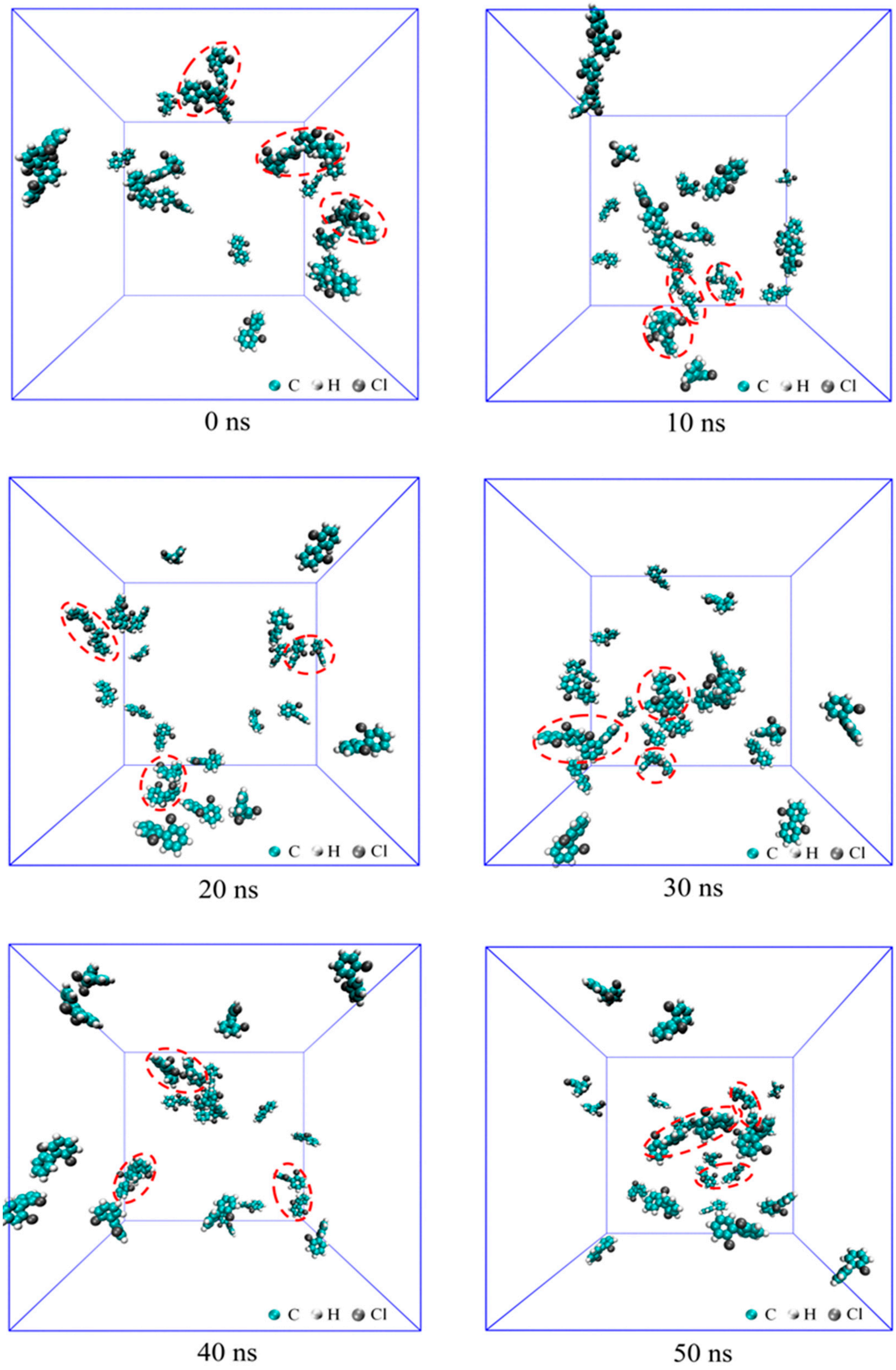

Figure S10. Changes of molecular conformation of PCB-4 at different simulation time (Red circles marked for dimers).

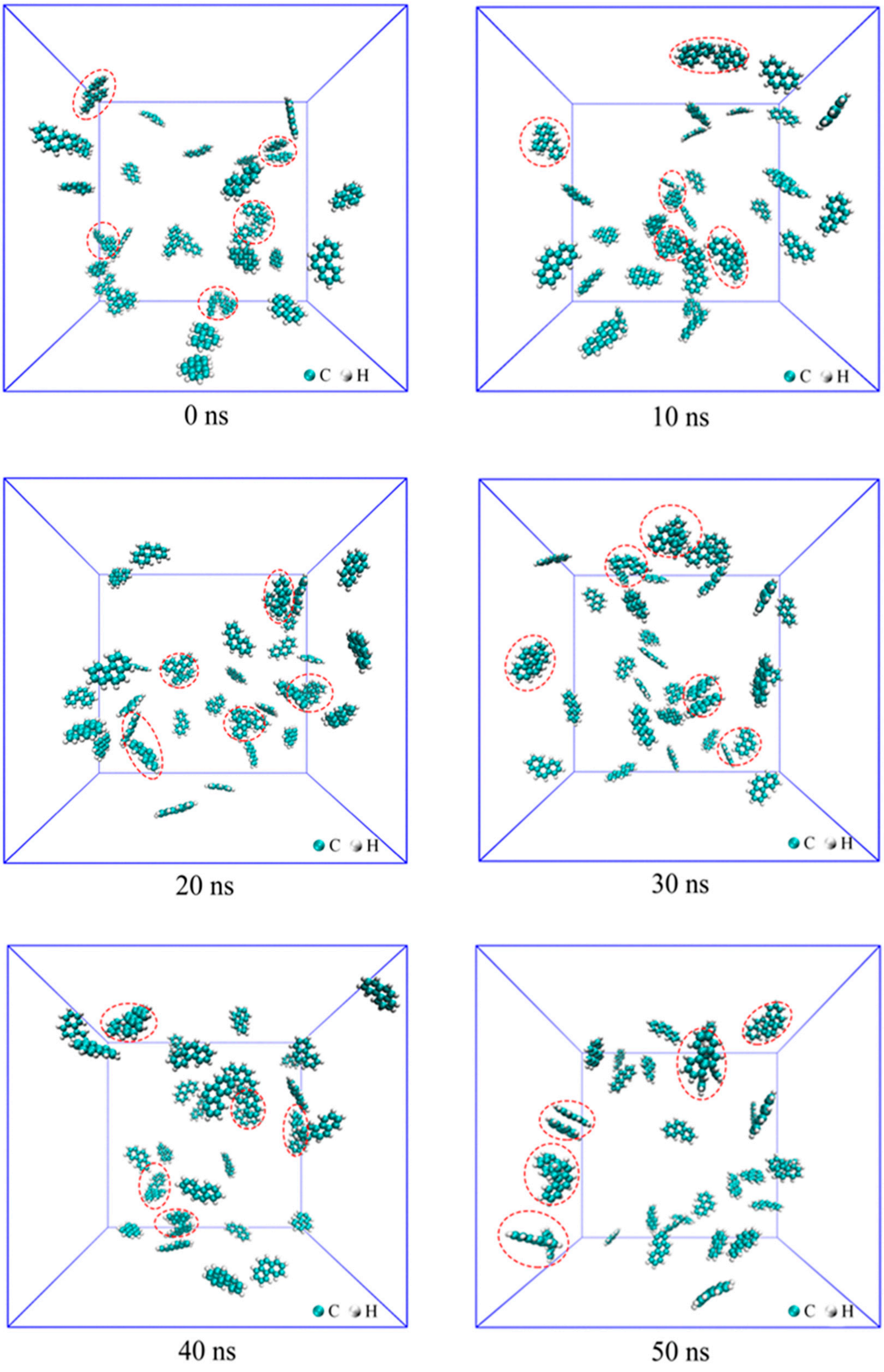

Figure S11. Changes of molecular conformation of Phenanthrene at different simulation time (Red circles marked for dimers).

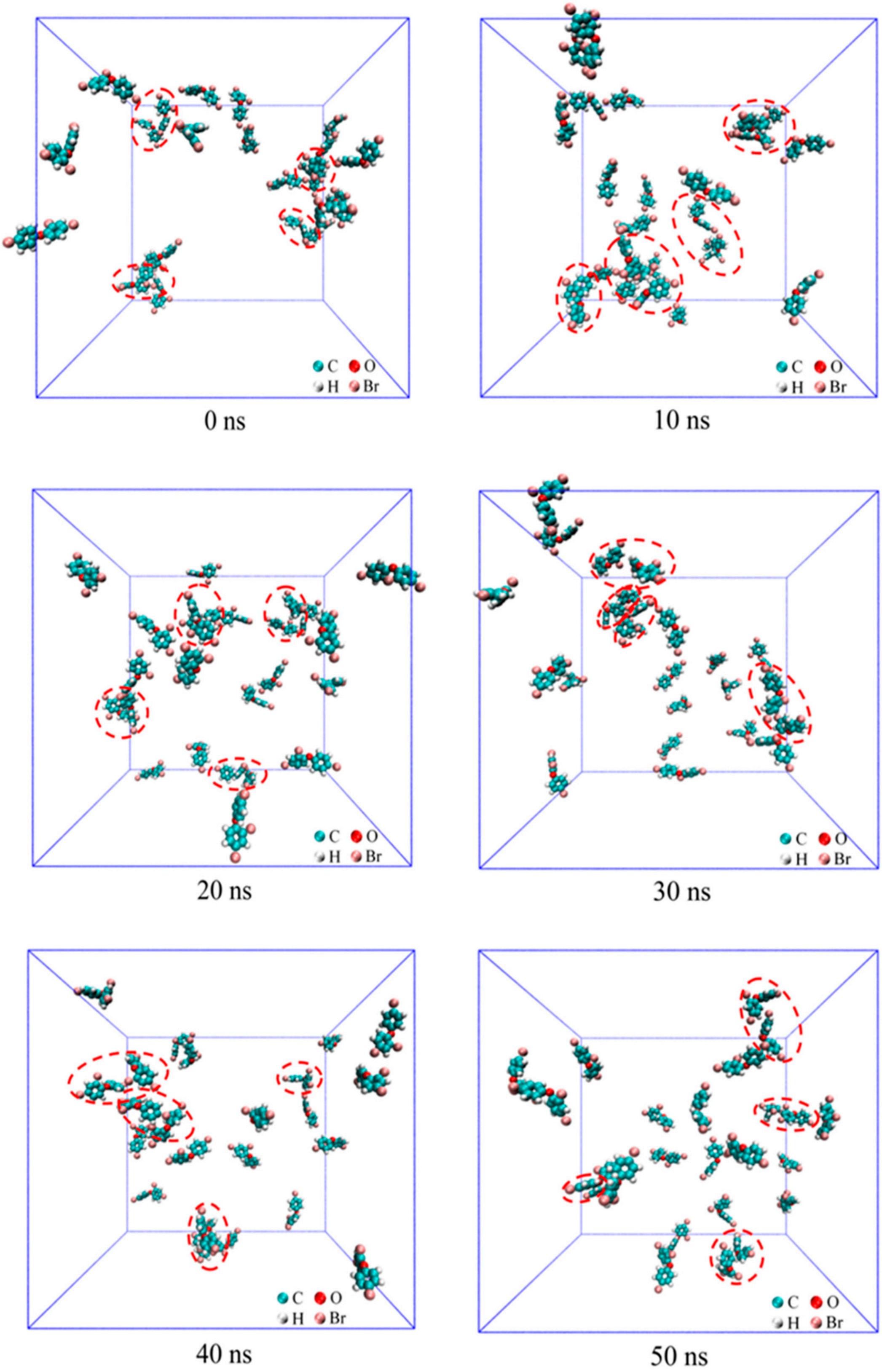

Figure S12. Changes of molecular conformation of PBDE-28 at different simulation time (Red circles marked for dimers).

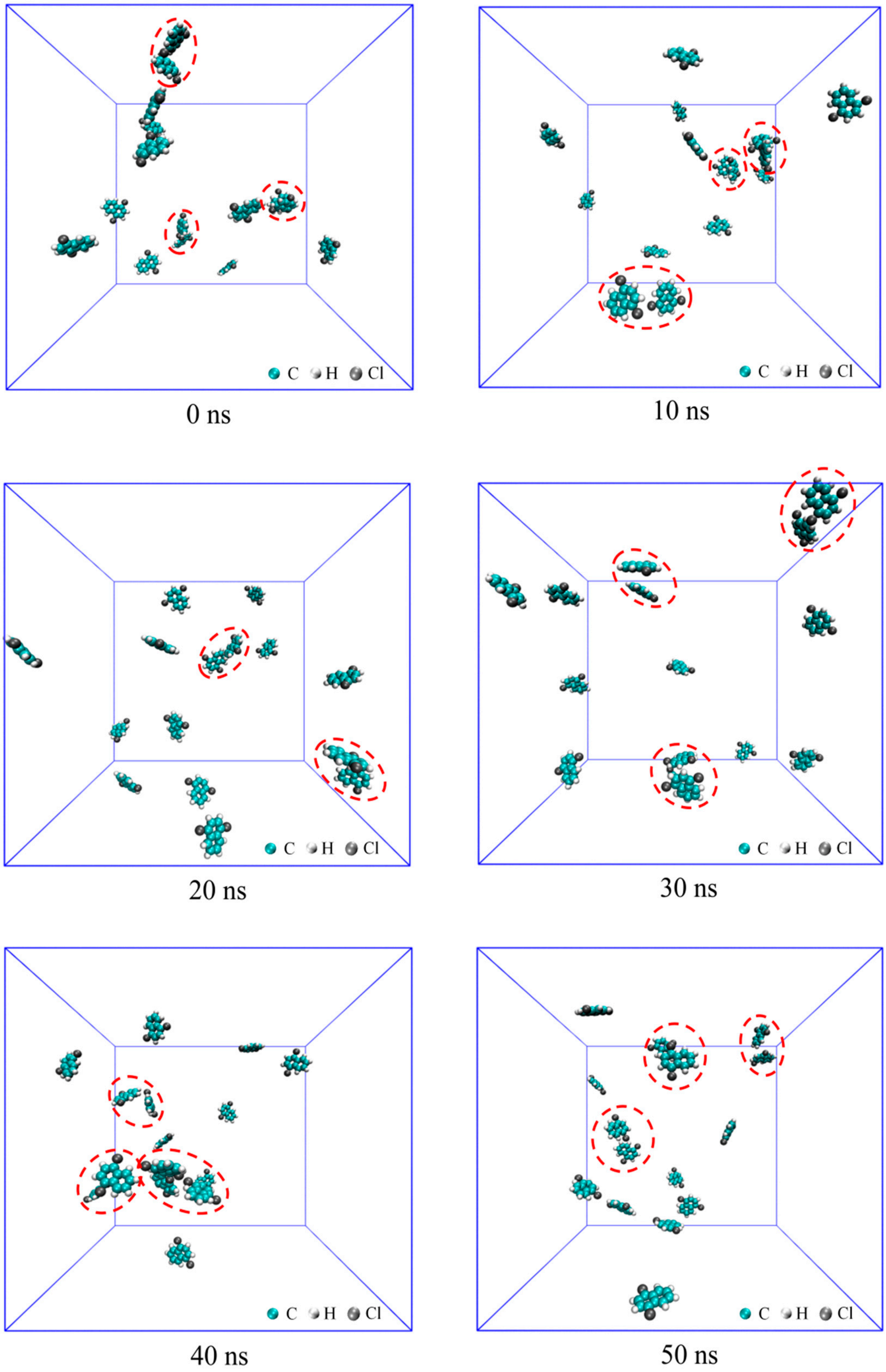

Figure S13. Changes of molecular conformation of PCN-5 at different simulation time (Red circles marked for dimers).

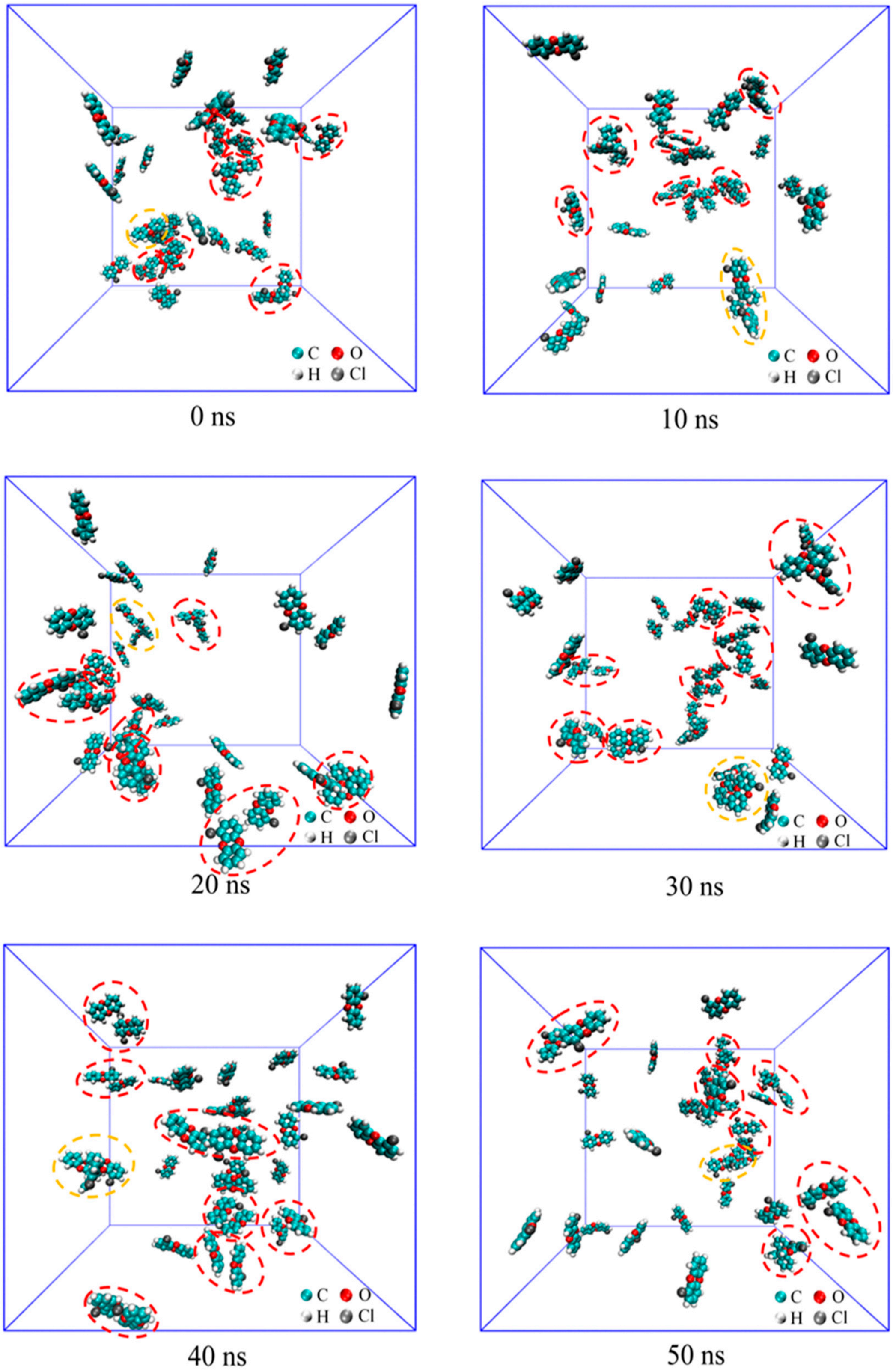

Figure S14. Changes of molecular conformation of PCDD-1 at different simulation time (Red circles marked for dimers and yellow circle for trimer).

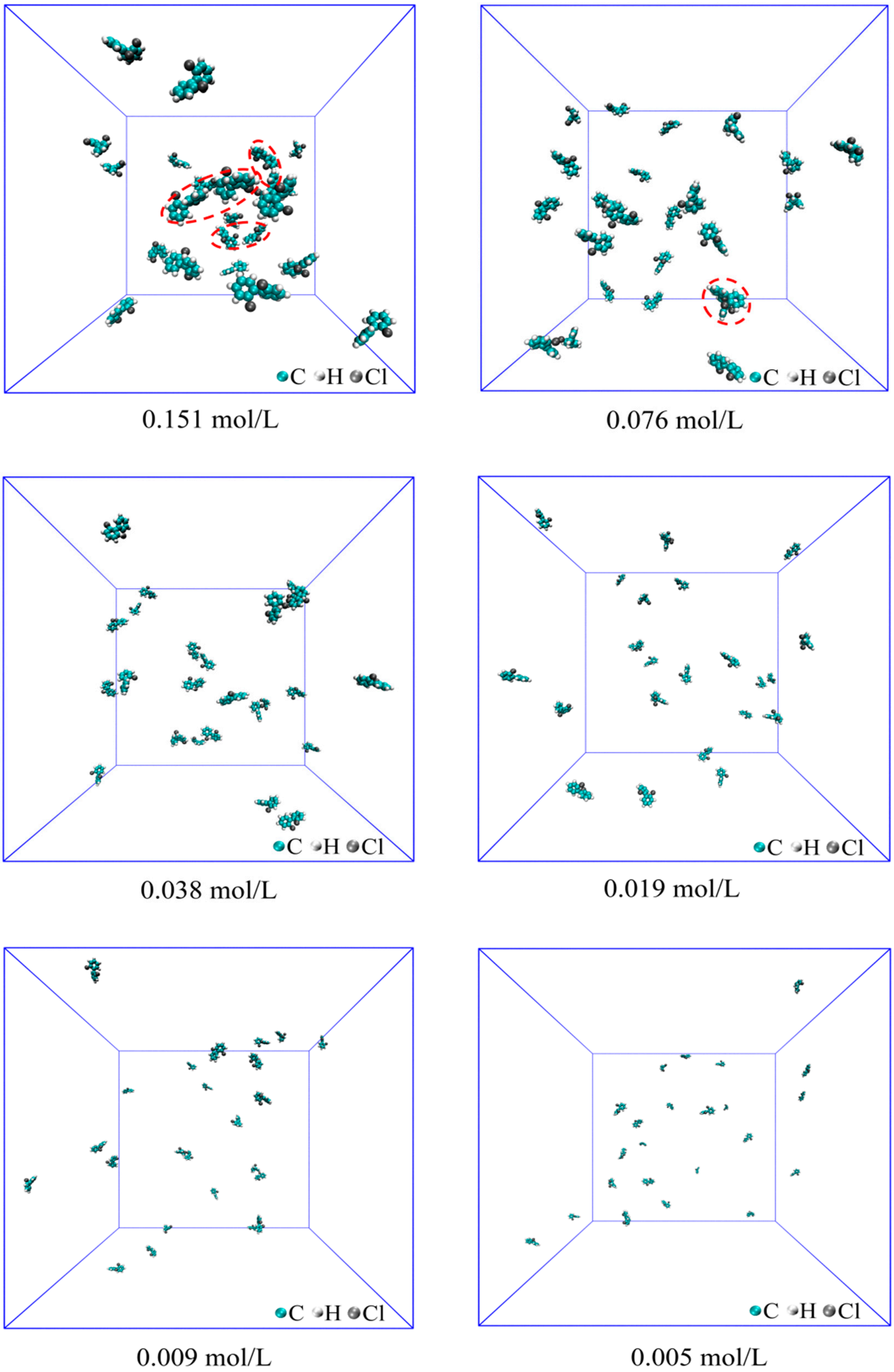

Figure S15. Molecular conformations of PCB-4 in the *n*-octanol phase at different concentrations (Red circles marked for dimers).

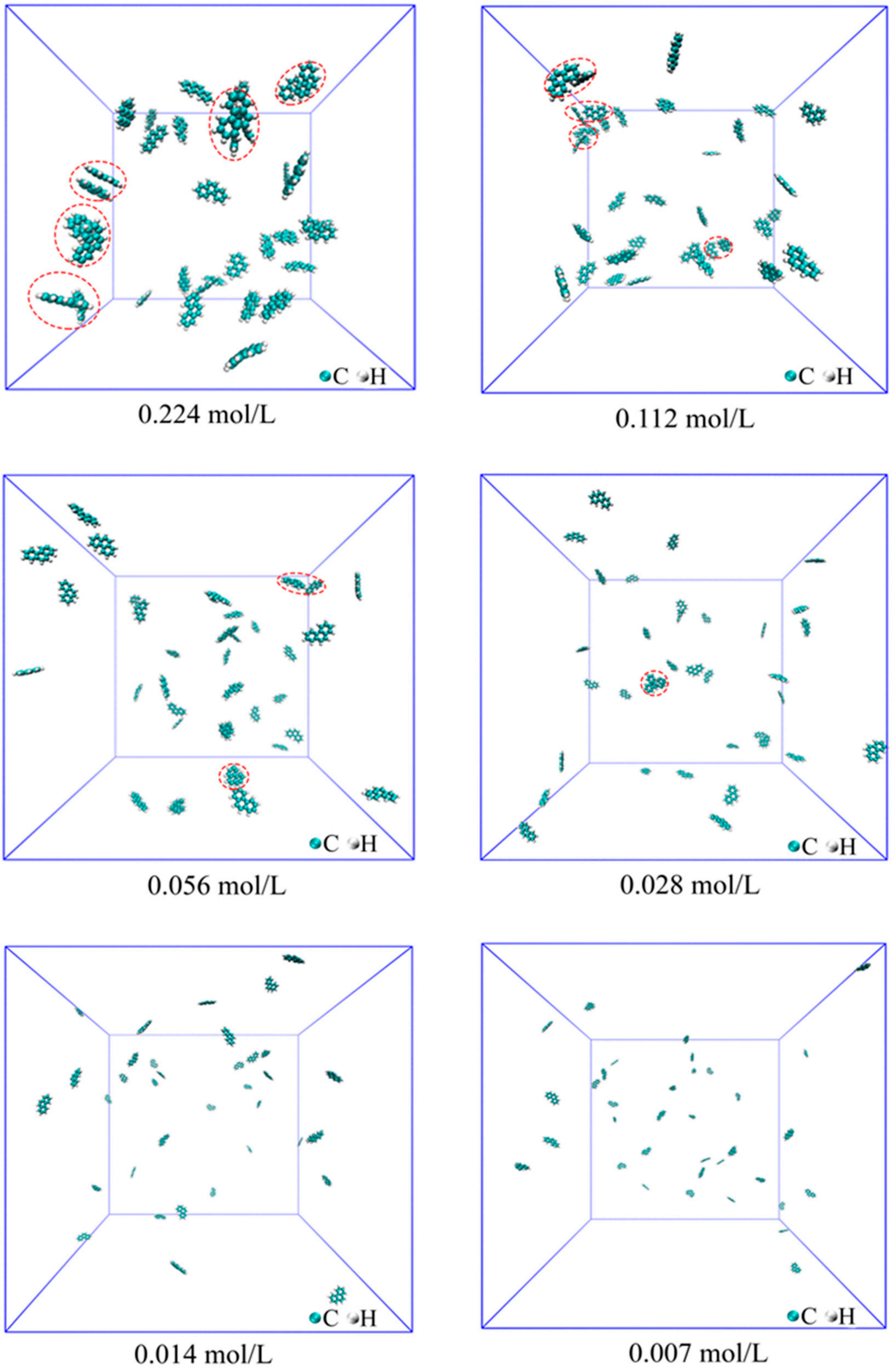

Figure S16. Molecular conformations of Phenanthrene in the *n*-octanol phase at different concentrations (Red circles marked for dimers).

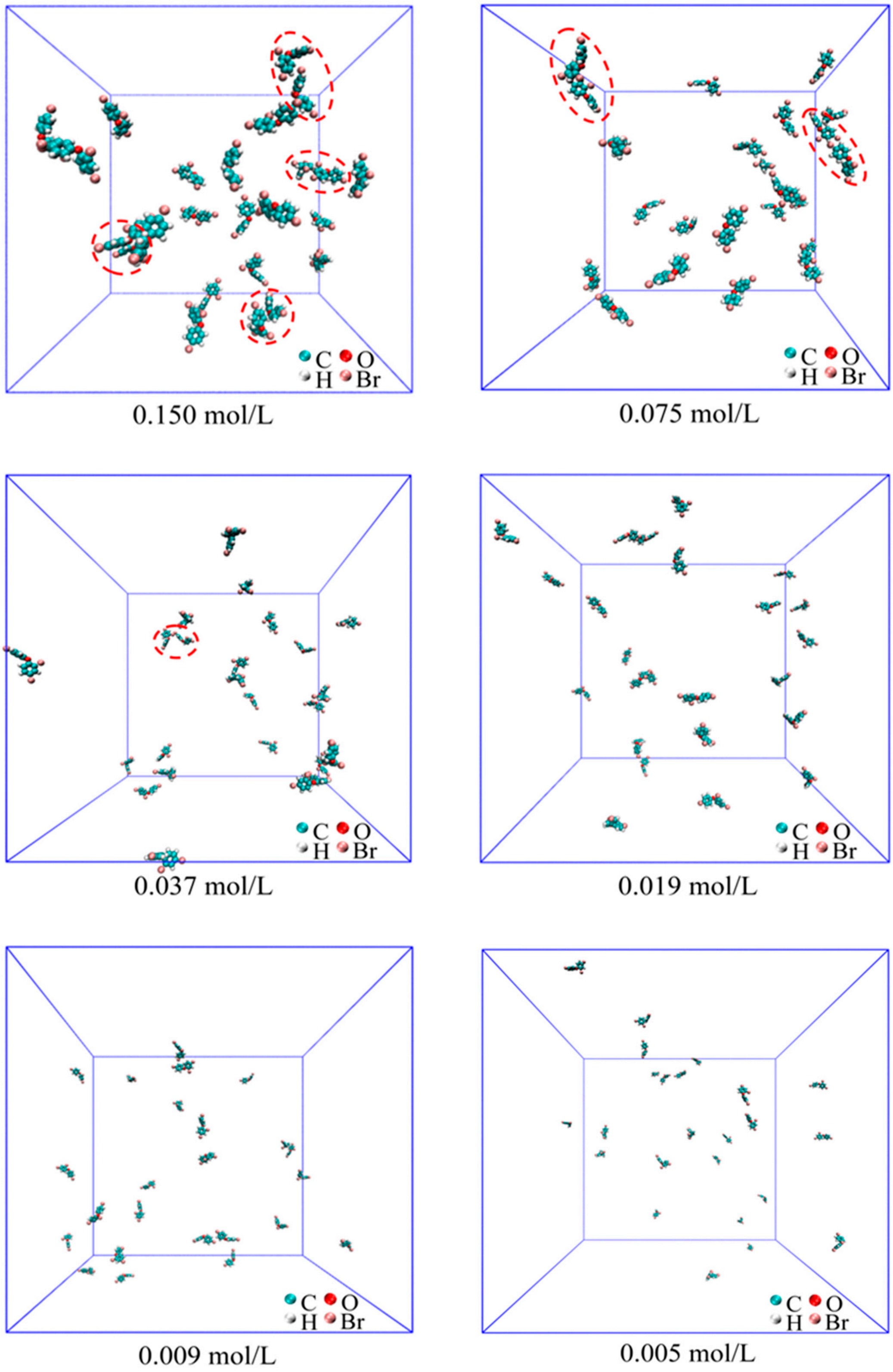

Figure S17. Molecular conformations of PBDE-28 in the *n*-octanol phase at different concentrations (Red circles marked for dimers).

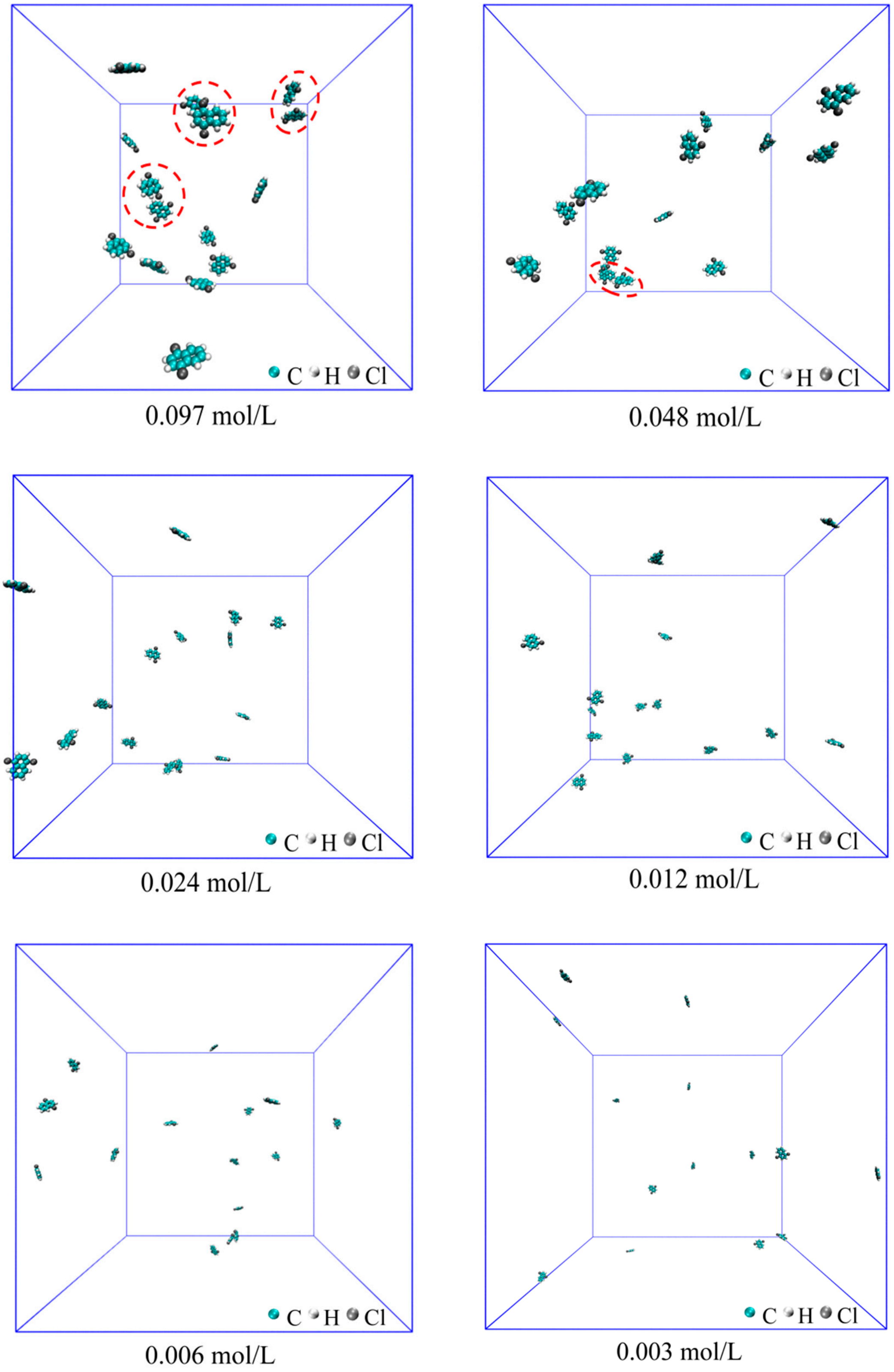

Figure S18. Molecular conformations of PCN-5 in the *n*-octanol phase at different concentrations (Red circles marked for dimers).

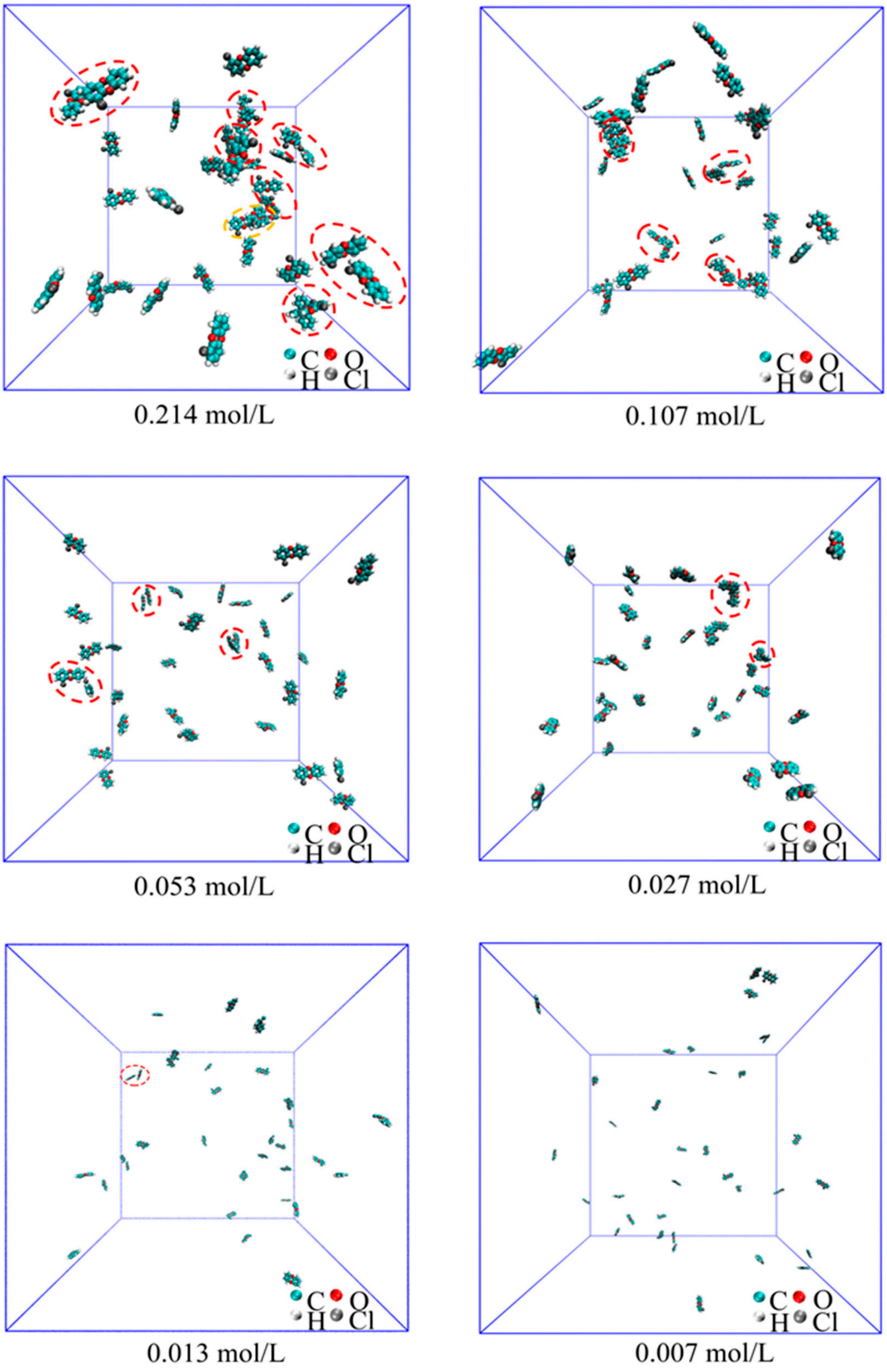

Figure S19. Molecular conformations of PCDD-1 in the *n*-octanol phase at different concentrations (Red circles marked for dimers and yellow circle for trimer).

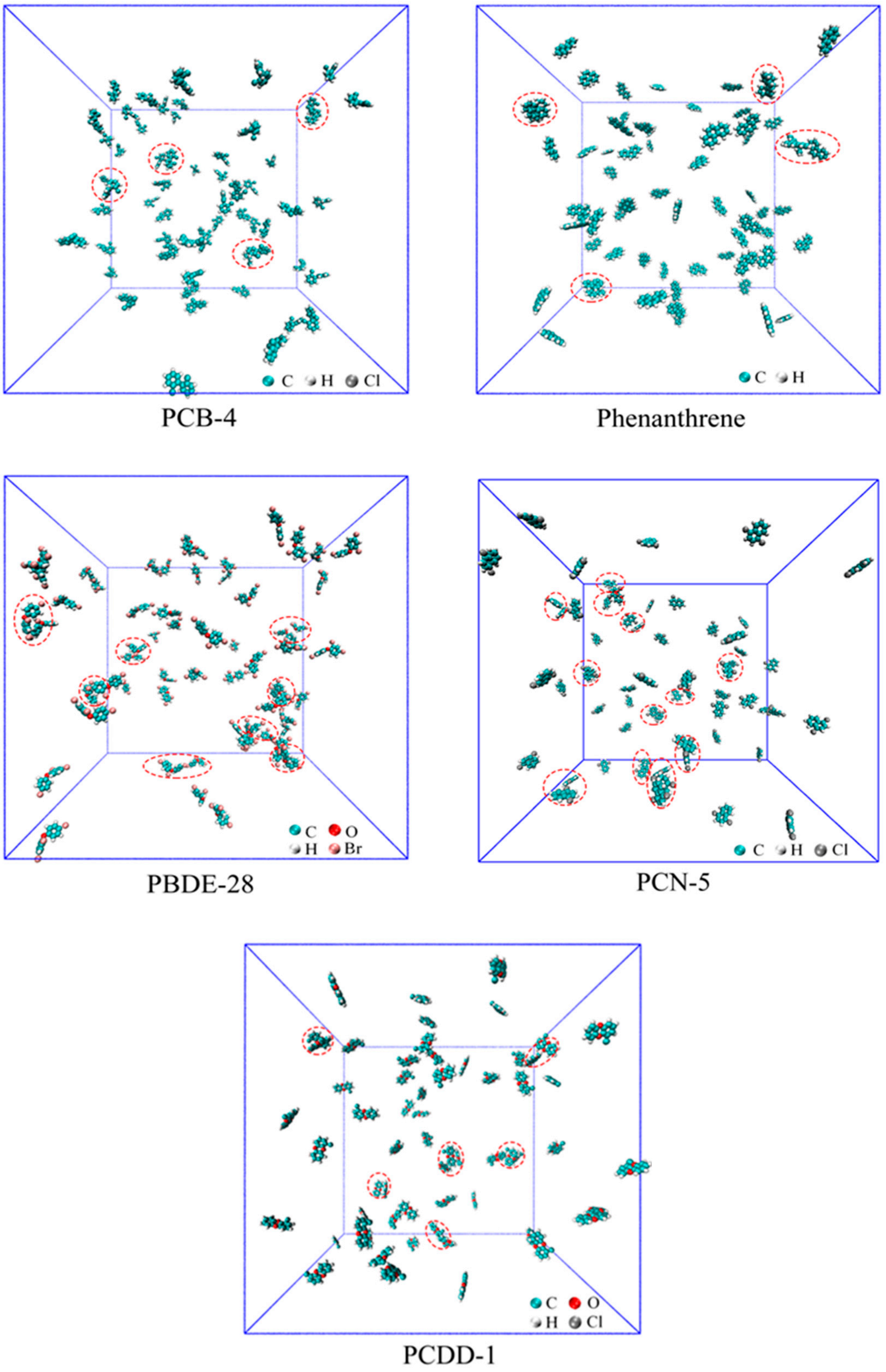

Fig. S20. Aggregation behavior of different aromatic pollutants in the *n*-octanol phase at the same concentration of 9.67×10^{-2} mol/L (Red circles marked for dimers).

Table S1. Molecular number of aromatic pollutants and *n*-octanol calculated at different concentrations.

**Concentration**	***n*(Chemical): *n*(Octanol)**				
	**PCB-4**	**Phenanthrene**	**PBDE-28**	**PCN-5**	**PCDD-1**
Saturation	24:1000	35:1000	24:1000	15:1000	34:1000
1/2 Saturation	24:2000	35:2000	24:2000	15:2000	34:2000
1/4 Saturation	24:4000	35:4000	24:4000	15:4000	34:4000
1/8 Saturation	24:8000	35:8000	24:8000	15:8000	34:8000
1/16 Saturation	24:16000	35:16000	24:16000	15:16000	34:16000
1/32 Saturation	24:32000	35:32000	24:32000	15:32000	34:32000

Table S2. Experimental and estimated log K_{OA} of 5 typical aromatic pollutants based on predicted aggregate percentages and their molecular structures.

**Chemicals**	**Experimental log K_{OA}**	**Predicted percentages (%)**			Estimated log K_{OA}	Molecular structure
		**Monomer**	Dimer	Trimer		
PCB-4	7.18	75.0	25.0	0	7.84	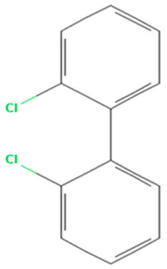
Phenanthrene	7.65	71.4	28.6	0	8.04	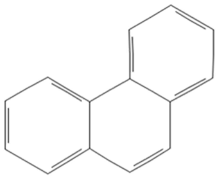
PBDE-28	9.50	66.7	33.3	0	9.61	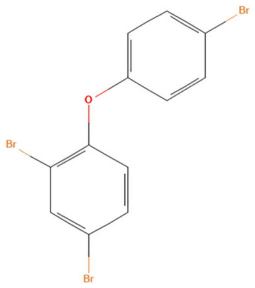
PCN-5	6.93	60.0	40.0	0	6.85	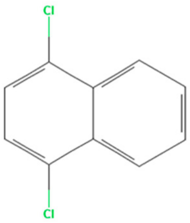
PCDD-1	7.86	50.0	41.2	8.8	7.54	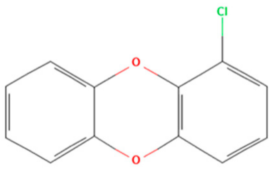

Table S3. Aggregate percentages, and experimental and estimated log K_{OA} values of PCB-4 at different concentrations.

Concentration (mol/L)	Monomer percentages (%)	Dimer percentages (%)	log K_{OA}	
			Experimental values	Estimated values
0.151	75.0	25.0	7.18	7.84
0.076	91.7	8.3		7.00
0.038	100.0	0		6.58
0.019	100.0	0		6.58
0.009	100.0	0		6.58
0.005	100.0	0		6.58

Table S4. Aggregate percentages, and experimental and estimated log K_{OA} values of Phenanthrene at different concentrations.

Concentration (mol/L)	Monomer percentages (%)	Dimer percentages (%)	log K_{OA}	
			Experimental values	Estimated values
0.224	71.4	28.6	7.65	8.04
0.112	77.1	22.7		7.72
0.056	88.6	11.4		7.09
0.028	94.3	5.7		6.77
0.014	100	0		6.45
0.007	100	0		6.45

Table S5. Aggregate percentages, and experimental and estimated log K_{OA} values of PBDE-28 at different concentrations.

Concentration (mol/L)	Monomer percentages (%)	Dimer percentages (%)	log K_{OA}	
			Experimental values	Estimated values
0.150	66.7	33.3	9.50	9.61
0.075	83.3	16.7		8.63
0.037	91.7	8.3		8.14
0.019	100.0	0		7.65
0.009	100.0	0		7.65
0.005	100.0	0		7.65

Table S6. Aggregate percentages, and experimental and estimated log K_{OA} values PCN-5 at different concentrations.

Concentration (mol/L)	Monomer percentages (%)	Dimer percentages (%)	log K_{OA}	
			Experimental values	Estimated values
0.097	60.0	40.0	6.93	6.85
0.048	86.7	13.3		5.59
0.024	100.0	0		4.96
0.012	100.0	0		4.96
0.006	100.0	0		4.96
0.003	100.0	0		4.96

Table S7. Aggregate percentages, and experimental and estimated log K_{OA} values of PCDD-1 at different concentrations.

Concentration (mol/L)	Monome percentages (%)	Dimer percentages (%)	Trimer percentages (%)	logK_{OA}	
				Experimental values	Estimated values
0.214	50.0	41.2	8.8	7.86	7.54
0.107	76.5	23.5	0		5.89
0.053	82.4	17.6	0		5.63
0.027	88.2	11.8	0		5.36
0.013	94.1	5.9	0		5.10
0.007	100.0	0	0		4.83
